# Identify potential drugs for cardiovascular diseases caused by stress-induced genes in vascular smooth muscle cells

**DOI:** 10.7717/peerj.2478

**Published:** 2016-09-28

**Authors:** Chien-Hung Huang, Jin-Shuei Ciou, Shun-Tsung Chen, Victor C. Kok, Yi Chung, Jeffrey J. P. Tsai, Nilubon Kurubanjerdjit, Chi-Ying F. Huang, Ka-Lok Ng

**Affiliations:** 1 Department of Computer Science and Information Engineering, National Formosa University, Yun-Lin, Taiwan; 2 Department of Bioinformatics and Medical Engineering, Asia University, Taichung, Taiwan; 3 Division of Medical Oncology, Kuang Tien General Hospital Cancer Center, Taichung, Taiwan; 4 School of Information Technology, Mae Fah Luang University, Chiang Rai, Thailand; 5 Institute of Biopharmaceutical Sciences, National Yang-Ming University, Taipei, Taiwan; 6 Department of Medical Research, China Medical University Hospital, China Medical University, Taichung, Taiwan

**Keywords:** Drug repositioning, Cardiovascular disease, Gaussian graphical model, Vascular smooth muscle cell, Gene set enrichment analysis, Topological parameters, Time-course microarray, Mechanical stress

## Abstract

**Background:**

Abnormal proliferation of vascular smooth muscle cells (VSMC) is a major cause of cardiovascular diseases (CVDs). Many studies suggest that vascular injury triggers VSMC dedifferentiation, which results in VSMC changes from a contractile to a synthetic phenotype; however, the underlying molecular mechanisms are still unclear.

**Methods:**

In this study, we examined how VSMC responds under mechanical stress by using time-course microarray data. A three-phase study was proposed to investigate the stress-induced differentially expressed genes (DEGs) in VSMC. First, DEGs were identified by using the moderated t-statistics test. Second, more DEGs were inferred by using the Gaussian Graphical Model (GGM). Finally, the topological parameters-based method and cluster analysis approach were employed to predict the last batch of DEGs. To identify the potential drugs for vascular diseases involve VSMC proliferation, the drug-gene interaction database, Connectivity Map (cMap) was employed. Success of the predictions were determined using in-vitro data, i.e. MTT and clonogenic assay.

**Results:**

Based on the differential expression calculation, at least 23 DEGs were found, and the findings were qualified by previous studies on VSMC. The results of gene set enrichment analysis indicated that the most often found enriched biological processes are cell-cycle-related processes. Furthermore, more stress-induced genes, well supported by literature, were found by applying graph theory to the gene association network (GAN). Finally, we showed that by processing the cMap input queries with a cluster algorithm, we achieved a substantial increase in the number of potential drugs with experimental IC50 measurements. With this novel approach, we have not only successfully identified the DEGs, but also improved the DEGs prediction by performing the topological and cluster analysis. Moreover, the findings are remarkably validated and in line with the literature. Furthermore, the cMap and DrugBank resources were used to identify potential drugs and targeted genes for vascular diseases involve VSMC proliferation. Our findings are supported by in-vitro experimental IC50, binding activity data and clinical trials.

**Conclusion:**

This study provides a systematic strategy to discover potential drugs and target genes, by which we hope to shed light on the treatments of VSMC proliferation associated diseases.

## Background

Cardiovascular diseases (CVDs), such as coronary heart attacks, arrhythmia, and cerebrovascular diseases (strokes), are the leading causes of death in many countries. According to the World Health Organization (WHO) report, CVDs affect tens of millions of human beings each year. Therefore, how to improve the diagnosis, treatment, and prevention of CVDs is an urgent and important issue. Vascular smooth muscle cells (VSMC) comprise the majority of the wall of the blood vessel and play an important role in the pathogenesis of CVD. It has been known that when the vessel is exposed to high pressure such as mechanical stretch, this injurious forces will lead to differential gene expression, and then induce VSMC hypertrophy and hyperplasia as well as changes in phenotype from the contractile state to the synthetic state and matrix proteins (Anwar et al., 2012; Intengan & Schiffrin, 2001; Levy et al., 1988). This type of transition between different phenotypes is referred to as “phenotypic modulation” or “vascular remodeling.” Contractile VSMC are elongated, spindle-shaped cells, whereas synthetic VSMC are less elongated and have a cobblestone morphology (Rensen, Doevendans & van Eys, 2007).

Vascular injury triggers VSMC dedifferentiation and then results in phenotypic modulation (Campbell & Campbell, 1990) which is the major cause of restenosis (Haudenschild, 1993), atherosclerosis (Rivard & Andres, 2000) and hypertension (Lemarié, Tharaux & Lehoux, 2010). However, to date, the intracellular molecular mechanisms that regulate the VSMC phenotype have not been well understood. In this study, we identified and characterized the mechanical stress-induced genes that resulted in an abnormal expression (namely, differentially expressed genes (DEGs)), as well as the inter-molecular interaction network in the VSMC.

Time-course gene expression experiments have been extensively used for studying a wide range of biological processes (BPs). Due to the use of the temporal structure embedded in the time-course data, we may be able to capture the dynamical behavior of the gene expression. In particular, time series data allow us to infer the gene association network (GAN).

To study how VSMC react in response to mechanical stress, time-course microarray data were used to identify stress-induced genes, and further to understand the role of gene regulatory networks under mechanical stress in the VSMC. First, we identified DEGs and then conducted the gene set enrichment analysis to highlight the most relevant biological process terms associated with a given gene list. Next, the Gaussian Graphical Model (GGM) was employed to infer the GAN by considering the partial correlation coefficient based on the previous study proposed by Schafer & Strimmer (2005). More studies had also reported the use of GGM in biological systems, such as, inferring the gene dependency network (Schafer & Strimmer, 2006), circadian rhythm regulatory network construction (Liu et al., 2012), and integration of multiple sources of genomics data to infer gene regulatory network (Chun et al., 2013). After that, the GAN derived from the GGM can be characterized by certain topological parameters. Finally, graph theory approach was introduced to analyze the GAN. Furthermore, we also validated that our prediction of stress-induced genes corresponds to previous publications; hence, we can demonstrate the effectiveness of the present approach.

## Materials and Methods

### Datasets

To examine how mechanical stress contributes to the response of VSMC and its underlying signal transduction pathways, we made use of the microarray experiment, E-MEXP-569, downloaded from the ArrayExpress database (Brazma et al., 2003). The experiment compared the gene expression profiles of the VSMC in response to a cyclical mechanical strain over a time-course of 0, 2, 4 and 24 h. Each sample consisted of two replicates prepared from human aortic VSMC purchased from Cambrex Bioscience.

### Differentially expressed gene identification

A gene which has an observed score that deviates significantly from the expected score is considered as a DEG. There are many statistical methods available for identifying DEGs by microarray data analysis. The use of false discovery rates (FDR) is a common approach to discover significant genes (Efron & Tibshirani, 2002). Analysis of variance (ANOVA) is another approach to investigate the impact of microarray gene expression values within a single factor (Kerr et al., 2002). Among the many statistical methods, Significance Analysis of Microarray (SAM) (Tusher, Tibshirani & Chu, 2001; Zhang, 2007), Empirical Bayes Analysis of Microarrays (EBAM) (Efron et al., 2001), and empirical Bayes statistics (EBAYES) (Efron, 2003) are three commonly employed approaches to identify DEGs.

SAM is a statistical method for identifying DEGs by comparing two or more groups of samples. It uses repeated permutations of the data to estimate FDR based on observed versus expected scores obtained from randomized data. SAM is able to handle time-course data, by considering different time points as distinct groups (Mutarelli et al., 2008).

EBAM performs one and two class analyses using either a modified t-statistic or standardized Wilcoxon rank statistic, and a multiclass analysis using a modified F-statistic. Moreover, this function provides an EBAM procedure for categorical data such as SNP data and the possibility of employing a user-written score function. EBAM can be applied to a differential time-course experiment to determine a subset of cDNAs that have the largest probability of being differentially expressed with respect to treatment conditions across a set of unequally spaced time points (Eckel et al., 2004).

The EBAYES algorithm computes moderated t-statistics, moderated F-statistics, and log-odds of differential expression by empirical Bayes shrinkage of the standard errors towards a common value. The moderated t-statistics is defined by

(1) $${\tilde t_{gk}} = {{{{\hat \beta }_{gk}}} \over {{{\tilde s}_g}\sqrt {{v_{gk}}} }}$$

where *g* and *k* denote the *g*th gene and the *k*th time respectively, the contrast estimator ${\hat \beta _{gk}}$ denotes the difference between two classes, ${\tilde s_g}$ stands for the shrinkage estimation of the standard deviation of the *g*th gene expression level, and ${v_{gk}}$ represents the *k*th diagonal element of the covariance matrix.

The EBAYES algorithm overcomes the possibility of identifying genes with small fold-change that might be significant from statistical analysis. In our previous study (Chen, Wu & Ng, 2012), we suggested that EBAYES, SAM, and EBAM achieve a similar level of cancer gene prediction accuracy, i.e. around 20%; therefore, without any bias EBAYES is adopted in the present analysis.

The microarray data were pre-processed by using the *Bioconductor* (Efron, 2003; Irizarry, 2005) R package ‘*limma*’ before passed to EBAYES for performing moderate F-test. The *p_EBAYES_*-value reported in the manuscript is the *p*-value for moderate F-test, i.e. *F.p.value* < 0.01, this value sets the threshold (Efron & Tibshirani, 2002) used to determine the DEGs.

### Gene set enrichment analysis

Functional annotation of the DEGs is given by implementing the Database for Annotation, Visualization and Integrated Discovery, DAVID (Huang, Sherman & Lempicki, 2009). DAVID accepts batch annotation and conducts GO term enrichment analysis to highlight the most relevant GO terms associated with a given gene list. The gene identifiers used in DAVID is the microarray probe ID, i.e. AFFYMETRIX_3PRIME_IVT_ID.

### Gaussian graphical model (GGM)

Inferring gene regulatory networks from microarray data is an important issue in systems biology. GGM is a graphical model, which was developed by Dempster (1972) to study the dependencies among a set of variables. In principle, the GGM infers GAN by considering the partial correlation coefficient instead of the Pearson correlation coefficient (PCC). The simple method of inferring GAN based on the PCC is not valid in most case studies because the high PCC of two variables does not imply a direct relationship. The GGM solves such a problem by using partial correlations to measure the independence of two genes. In partial correlation calculation, one introduces a third variable that has a relationship between the other two variables, and then calculates the correlation between two variables while excluding the impact of the third variable. Therefore, GGM allows us to distinguish between direct and indirect gene-gene interactions.

Within the GGM framework, the presence of an edge between two genes, *g_i_* and *g_k_*, is determined by the value of the partial correlation, *pcor(i,k)*. Given the covariance matrix of all genes, Σ, it can be shown that full order *pcor(i,k)* is given by (Schafer & Strimmer, 2005),

(2) $$pcor(i,k) =-{{{\omega _{ik}}} \over {\sqrt {{\omega _{ii}}{\omega _{kk}}} }},$$

where ${\Sigma ^{-1}} = {\omega _{ik}}$, which denotes the covariance between the i-th and j-th genes in the inverse of the covariance matrix. If an entry in the inverse covariance matrix is close to zero, then genes *g_i_* and *g_k_* are condition independent given all remaining genes. Since the number of microarray samples is much smaller than the number of genes considered, we employed a technique called shrinkage to improve the estimation of the sample covariance matrix. In actual implementation, we used the R package, *GeneNet* (Schäfer, Opgen-Rhein & Strimmer, 2006) to infer the GAN from microarray data.

### Topological graph theory

In this work, we introduce the graph theory approach to analyze the GAN. Many studies indicated that there are underlying global and local topological structures of biological networks. The GAN derived from the GGM may have a complex topology. A complex network can be characterized by certain topological parameters; these parameters can be computed by using the SBEToolbox (Konganti et al., 2013). The 11 computed topological parameter values have been normalized between −1 and 1, a larger topological parameter value implies stronger topological effect. Three global topological parameters (average graph distance, diameter and network efficiency) and eight local topological parameters; i.e. the topological parameters of a node in the network (closeness centrality (CC), degree centrality (DC), eccentricity centrality (EC), betweenness centrality (BC), bridging centrality (BRC), clustering coefficient (CLC), brokering coefficient (BROC), local average connectivity (LAC)) are defined in the Section 1 of the Supplemental Information.

In the previous study, we have proposed a method to identify the important nodes in a network by topological parameter-based classification (Huang, Chen & Ng, 2016). Given a GAN, we propose that nodes have a higher degree, betweenness, centrality and densely connected which may play an important role in VSMC under mechanical stress. To test and demonstrate the presented concept, we classified eight of the 11 parameters into five groups based on their topological properties. Our classification considers the local parameters only. Classification of the eight parameters is shown in Table 1. Global topological parameters are not included, i.e. diameter, average graph distance and network efficiency.

**Table 1:** Classification of the local topological parameters.

Group	Topological parameter	Abbreviation
1	Degree centrality	DC
2	Betweenness centrality	BC
3	Bridging centrality	BRC
4	Closeness centrality, eccentricity centrality	CC, EC
5	Clustering coefficient, brokering coefficient, local average connectivity	CLC, BROC, LAC

In order to further our understanding of the GAN network property, we sorted the results of the eight local parameters’ values in descending order. For each group, if a node is ranked in the top 10%, a score value (SV) of one is assigned to this node. Since there are five groups, a node can have a maximum score of five. Since groups 4 and 5 consist of more than one topological parameters, a node will receive an SV of one if it ranks in the top 10% in any one of the parameters. The same analysis is repeated with the 15 and 20% thresholds.

### Cluster analysis by using CFinder

In order to deepen our understanding of the GAN, we proposed that genes do not highly interact with others were assumed less important and consequently removed before the enrichment analysis.

The clustering tool, CFinder (Palla et al., 2005) which is based on the clique percolation clustering approach, was employed to perform the cluster analysis. The CFinder program identifies interacting clusters, which are called *k*-communities. A 3-community is set as *k* being equal to three (complete subgraphs of size 3). Any two *k*-communities are adjacent if they share *k*−1 common nodes. A *k*-community is constructed by merging all possible adjacent (*k*−1)-communities.

### Drug repositioning

The idea of drug repositioning is a recently developed approach in the pharmaceutical industry that endeavors to identify new uses for existing drugs; and has achieved certain successes (Ashburn & Thor, 2004). This approach has the potential to reduce the development time required for drug discovery, as well as reducing side-effects. There are many works on identifying repositioned drugs which are based on various methods: the graph-based inference method (Iorio et al., 2010; Wu, Wang & Chen, 2013), the microarray expression method (Wu, Wang & Chen, 2012), and the differential expressed correlation method (Sun et al., 2013).

We made use of the drug-gene interaction databases, Connectivity Map (cMap) (Lamb, 2007), to find potential drugs for the treatments of VSMC proliferation associated diseases. Although the cMap resource is aimed at identifying drug treatment for cancer diseases, it is hypothesized that some of the known drugs may be repositioned for treating VSMC proliferation associated diseases.

Both CVDs, including atherosclerosis, as well as cancers lead to the cause of death worldwide, and they are characterized by a local increase in tissue mass. Many studies have suggested that atherosclerosis and cancer formation involve similar cellular processes i.e. cell proliferation, inflammation, and genomic instability. Both types of diseases possess a significantly common role in the pathogenesis and progression of atherosclerosis and cancer, especially in molecular pathways (Li & Gao, 2005; Ross et al., 2001a; Ross et al., 2001b). Common pathways or signal transduction networks, such as PI3k/Akt, can mediate several functional and morphological alterations of VSMC after being activated to develop vascular diseases (Jung et al., 2000), as well as affect the growth, apoptosis and cell cycle regulation of various cell types to induce cancer progression (Arcaro & Guerreiro, 2007). It is also known that the MAPK pathway, involved in VSMC proliferation, hypertrophy, and migration, central to the pathogenesis of vascular diseases (Jacob et al., 2005), possessed the same effects similar to PI3k/Akt in cancer occurrence (Sebolt-Leopold & Herrera, 2004).

Based on the above observations, we put forward the hypothesis for further investigation. It is conjectured that a drug molecule may potentially reverse the CVDs signature if the molecule induced signature is significantly negatively correlated with the disease-induced signature found in the cMap.

Since the time-course expression profile of DEG may exhibit an oscillatory pattern, therefore, we filtered out up- and down-regulated DEGs with at least two consecutive time points are up and down regulated respectively.

This set of DEGs were used to query the cMap database, where potential drugs with adjusted *p_cMAP_*-value less than 0.05 are retained. The adjusted *p_cMAP_*-value (named permutation *p-*value) is an estimate of the likelihood that the enrichment of a set of potential drugs in the list of all cMap drugs in given result/prediction would be observed by chance. This permutation *p*-value is determined by computing the enrichment of 100,000 sets of potential drugs selected at random from the set of all cMap drugs in the result/prediction.

There may be concern that the times points 0, 2, 4, 24 h are not consecutive. As Qiu et al. (2014a) noted in their review, mechanical stress regulates the functions of VSMC within 24 h in most of the studies (for details of the study, see the Table 1 (12 studies) and Table 2 (21 studies) of the review article by (Qiu et al., 2014a). For instance, Feng et al. (1999) reported that the gene expression fold changes of mechanically induced genes at 12 and 24 h were remarkably similar except only for three genes out of 3,160 DEGs.

**Table 2:** The 23 DEGs, their functional description and supporting study.

Gene	Full gene name	Functional description	Study
*CCL2*	Chemokine ligand 2	Aged VSMC exhibited upregulation of CCL2 gene	Mascall et al. (2012) and Song et al. (2012)
*CDC*	Cell division cycle proteins	Involved in controlling cell cycle phase transition, such G2/M transition in VSMC	Su et al. (2007)
*CDH6*	Cadherin 6	A VSM cell-cell adhesion molecule, it is important for tissue integrity and cell proliferation	Sun et al. (2014)
*CDK*	Cyclin-dependent kinase	Differential effects on VSMC proliferation	Tanner et al. (2000)
*CEBPD/GADD*	Growth arrest and DNA damage-inducible gene	Cyclic stretch enhanced GADD153 expression in cultured rat VSMC	Oyadomari & Mori (2004) and Cheng et al. (2008)
*COL*	Collagen	Is expressed by VSMC in response to vascular injury	Sibinga et al. (1997)
*CTGF*	Connective tissue growth factor	Stimulates VSMC growth and migration	Fan & Karnovsky (2002) and Fan, Pech & Karnovsky (2000)
*CXCL2*	Chemokine (C-X-C Motif) ligand 2	Aged VSMC exhibited upregulation of CXCL2 gene	Song et al. (2012)
*HSP*	Heat shock protein	Cyclic strain-induced expression of HSP in human endothelial cells	Hurley et al. (2010)
*IGF1R*	Insulin-like growth factor type 1 receptor	Mechanical stretch simulates proliferation through activation of IGF1R	Cheng & Du (2007), Li et al. (2008), Okura et al. (2001) and Qiu et al. (2014b)
*IL*	Interleukin-1	Plays a role in the migration of VSMC into the neointima following acute injury	Dardik et al. (2005), Wójtowicz et al. (2010) and Zampetaki et al. (2005)
*JUNB/AP-1*	Jun B proto-oncogene	Is rapidly activated in a balloon-injured artery in rat	Yasumoto et al. (2001)
*MAPK*	Mitogen-activated protein kinase	Mechanical stress-initiated MAPK signal transduction in VSMC	Li et al. (2000) and Li & Xu (2000)
*MCM3AP*	Minichromosome maintenance complex component 3	Expression of minichromosome maintenance proteins in VSMC	Bruemmer et al. (2003a) and Bruemmer et al. (2003b)
*MMP*	Matrix metalloproteinase	Upregulation of MMP-1 plays a critical role in the flow-enhanced motility in VSMC	Asanuma et al. (2003), Chung et al. (2005), Feng et al. (1999), Pascarella et al. (2008) and Shi et al. (2009)
*PCNA*	Proliferating cell nuclear antigen	Cyclic strain-induced expression of PCNA in human endothelial cells	Hurley et al. (2010) and Richard et al. (2007)
*PDGF*	Platelet-derived growth factor	Plays a role in the migration of VSMC into the neointima following acute injury	Dardik et al. (2005) and Raines (2004)
*SGK1*	Serum-glucocorticoid-regulated kinase1	Mechanical stress-activated SGK1 contributes to neointima formation in vein graft	Cheng et al. (2010)
*TENM*	Tenascin	Mechanically induced genes in human VSMC	Feng et al. (1999)
*TGF*	Transforming growth factor	Involved in controlling cell cycle phase transition, such G2/M transition in VSMC	Su et al. (2007), Mata-Greenwood et al. (2005), Reddy & Howe (1993) and Ueba, Kawakami & Yaginuma (1997)
*TIMP*	TIMP metallopeptidase inhibitor 3	Mechanical strain induces TIMP-1 and TIMP-2 synthesis. Inhibition of angiogenesis in human artery endothelial cells is mediated by TIMP-2 from VSMC	Chung et al. (2005) and Mascall et al. (2012)
*TNF*	Tumor necrosis factor	Induction of MMP-14 and 2 by cyclical mechanical stretch is mediated by TNF	Wang et al. (2003)
*TP53*	p53 protein	Plays a role in VSMC proliferation and atherosclerosis	Mercer & Bennett (2006) and Yonemitsu et al. (1998)

We further noted that the use of non-consecutive time points for gene expression measurements are rather common; for example, Morrow et al. (2005) examined the effect of strain at the 6, 10 and 24 h, whereas Schad et al. (2011) investigated the strain effect at the 1, 6, 18 and 48 h.

Cell viability was determined using the MTT and clonogenic assay. Cancer cell lines, A549 and H460 were used in both experiments. The protocols were described in our previous works (Huang et al., 2014; Lan et al., 2010). Furthermore, we also submitted the predicted DEGs to NCBI PubChem database and the DrugBank databases (Wishart et al., 2008) to identify potential drugs for VSMC proliferation associated diseases. The results of the potential drugs identified by cMap are reported in the following ‘Results’ section.

IC50 is the drug concentration inducing 50% inhibition of the cell viability. Cell viability was determined using the MTT and clonogenic assay. Non-small cell lung cancer cell lines were used in both experiments. In this paper we retrieved two IC50 measurements from two different resources to validate our predictions, for clarity, “IC50” and “IC_db_50” represent experimentally determined IC50 activities obtained from in-house measurement (MTT and clonogenic assay) and the NCBI PubChem database, respectively.

## Results

To determine the DEGs, we made use of the “Linear Models for Microarray Data” (*limma*) package, which is available from the *Bioconductor* service. Details are described in the following sections.

### Differentially expressed gene identification

In this study, the Robust Multi-array Average (RMA) was used for gene expression normalization. After that, a model matrix (use the function, *model.matrix*) was created with rows and columns denoting the replicates (with the four time points information) and the time points respectively. Then, we seek a linear model to describe each probe/gene using the *lmFit* function provided by the *limma* package (Smyth, 2004; Smyth, 2005). DEGs are determined by first constructing the contrast matrix (use the function, *cont.matrix*), which made a pairwise comparison of two consecutive time points between the two replicates (use the function *contrast.fit*). EBayes analysis was subsequently conducted on the previous results, and the DEGs were selected by setting a *p_EBAYES_*-value threshold of 0.01 corresponding to the moderated *F*-statistics.

Through a manual literature search, we have collected a list of genes that are involved in VSMC phenotypic modification. Among the 473 DEGs, there are at least 23 DEGs are found in the literature. Table 2 summarized the results of those 23 genes, their biological functions, and references. These results of the DEGs can be accessed at http://ppi.bioinfo.asia.edu.tw/vsmc, which provides several important types of genetic information, such as the chromosomal locations, PubMed IDs, GO annotations, pathways, and *p_EBAYES_*-values.

### The results of gene set enrichment analysis

A total of 473 DEGs were submitted to DAVID for clustering, and thus enriched BPs related gene groups were obtained, included KEGG, BioCarta and REACTOME, in which not only the most often found enriched BPs are cell cycle-related processes, but also the enriched pathways are associated with cancer- or cell cycle-related events. These results show that many DEGs might involve in the cell proliferation or apoptosis regulatory pathways to induce cell proliferation or apoptosis which lead to cardiovascular disease or cancer.

Details of the top three clusters (with enrichment scores 13.84, 8.59, and 5.93 given by DAVID) of enriched BPs information were presented in Table 3. Obviously, the most often found enriched BPs are cell-cycle-related processes, such as, M phase, regulation of mitotic cell cycle and cytoskeleton organization. For the cluster with the highest enrichment score, 64 genes were included in the cell cycle process.

**Table 3:** The Results of the top three clusters’ enriched BPs.

Enrichment score: 13.84	Count	*p_DAVID_*-value	Benjamini
Cell cycle	64	6.90E-17	2.60E-13
M phase	41	9.30E-17	1.30E-13
Organelle organization	86	4.00E-16	3.40E-13
Cell cycle process	52	1.60E-15	9.70E-13
Cell cycle phase	44	2.40E-15	1.10E-12

Enrichment score: 8.59	Count	*p_DAVID_*-value	Benjamini
Regulation of cell cycle	36	6.80E-13	1.60E-10
Regulation of mitotic cell cycle	19	5.50E-08	7.10E-06
Cell cycle checkpoint	14	4.50E-07	5.50E-05

Enrichment score: 5.93	Count	*p_DAVID_*-value	Benjamini
Cytoskeleton organization	31	6.90E-07	7.60E-05
Microtubule cytoskeleton organization	17	9.60E-07	1.00E-04
Spindle organization	10	1.50E-06	1.50E-04
Microtubule-based process	22	1.90E-06	1.80E-04

**Note:**

The ‘Count’ denotes the number of DEGs found in the BPs. The ‘*p_DAVID_*-value’ and ‘Benjamini’ columns denote the *p*-value and Benjamini *p*-value given by DAVID, respectively.

Many studies have noted that hemodynamic factors, including shear stress, cyclic strain, and hydrostatic pressure, can (i) regulate the proliferation rate of VSMC (Boehm & Nabel, 2001; Sterpetti et al., 1993; Sterpetti et al., 1992), (ii) and thus trigger many cell-cycle-related molecules (Qiu et al., 2014a; Shi & Tarbell, 2011), and then (iii) decrease proliferation and increase apoptosis, mediated by the Akt pathway (Fitzgerald et al., 2008).

For the cluster with the highest enrichment scores, 64 genes were included in the cell cycle process. It has been reported that cell cycle and cell migration proteins regulate multiple biological functions in the cardiovascular systems (Boehm & Nabel, 2001).

For the M phase process, there are 41 genes. A number of studies have shown that the transition of VSMC from G2 phase into the M (mitosis) phase of the cell cycle is a tightly controlled process (Grainger et al., 1993; Somoza et al., 2004; Su et al., 2007). Furthermore, much evidence (de la Cuesta et al., 2013; Hellstrand & Albinsson, 2005; Martinez-Lemus, 2014; Sarkar et al., 2005; Zheng et al., 2010) also suggested that cytoskeleton deregulation may explain how VSMC switch from a contractile to a synthetic phenotype.

### Topological analysis of the gene association network (GAN)

**Figure 1:**
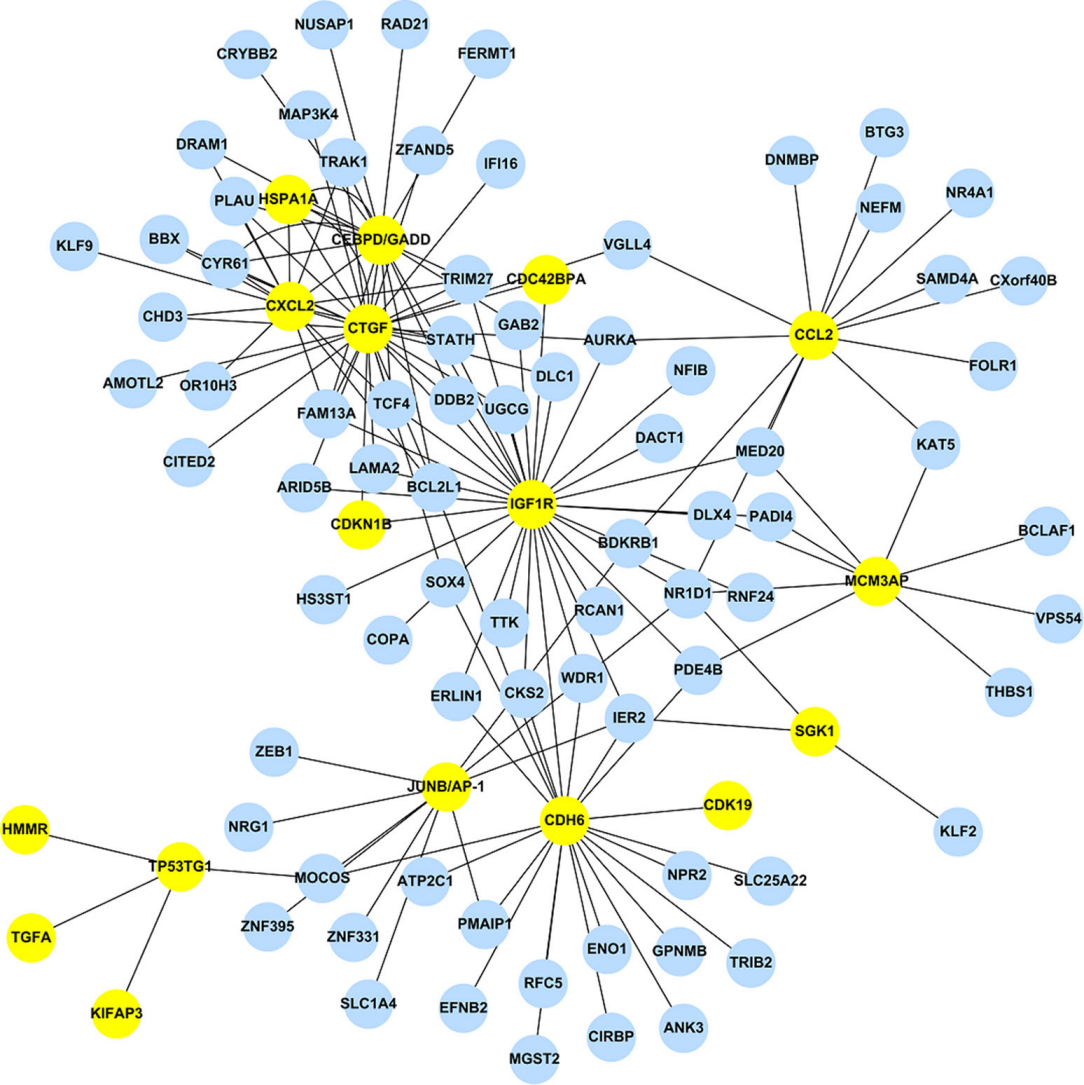
The stress-induced GAN for the 17 DEGs are shown by the yellow circles. Blue circles denoted DEGs, which were predicted by EBayes analysis.

Using the 473 DEGs as input, we performed the GGM analysis, and ranked the partial correlation coefficients according to their absolute values, i.e. both activation and suppression events were kept. After ranking the results obtained from GGM, only the top 1% correlation events were selected to construct the GAN. GGM analysis reduced the total number of DEGs from 473 to 243. This network consists of the 1,442 significant links (adjusted *p_GGM_*-value < 0.01) among the 243 genes. Among those 243 genes, 17 genes are the DEGs listed in Table 2, while the corresponding GAN is shown in Fig. 1 using Cytoscape (http://www.cytoscape.org/), a useful tool for visualizing a molecular interaction network. The 17 genes were highlighted and their linked genes (each link has an adjusted *p_GGM_*-value < 0.01) were shown. The R package, *network.test.edges*, was used for assessing the significance of edges in the GGM. A mixture model is fitted to the partial correlations using the R language false discover rate tool, *fdrtool*. This results in two-sided *p-*values for the test of non-zero correlation.

The top 1% correlation events obtained from the GGM analysis has a modest percentage of overlapping genes, i.e. 73.9% (17/23), where the rest of the 226 genes are DEGs but not included in Table 2.

Given the GAN (1,442 links among 243 genes), we analyzed the network by using the topological graph theory approach. The results of the 11 parameters of the GAN were summarized in Table 4.

**Table 4:** The results of global and local topological parameters of the GAN.

	Average	Standard deviation
Graph distance	2.68	–
Graph diameter	6.00	–
Network efficiency	0.41	–
Degree centrality	0.05	0.049
Betweenness centrality	0.007	0.015
Bridging centrality	0.0002	0.00027
Closeness centrality	0.38	0.054
Eccentricity centrality	0.23	0.0285
Clustering coefficient	0.50	0.331
Local average connectivity	0.372	0.281
Brokering coefficient	−0.33	0.241

In Table 4, it was found that the GAN had an average node graph distance of 2.68, and a network diameter of 6. A small average node graph distance value means that any two genes may have indirect interactions via less successive gene-gene interaction.

Using 0.05 as a threshold, the VSMC GAN had a relatively small *DC* value, i.e. 0.05. This means that nodes of GAN were much less interaction *directly* with each other. Here ‘*direct*’ refers to the fact that *DC* characterized a node’s neighborhood connections. However, the GAN had a rather small *BC* (0.007) and *BRC* (0.0002) values. A higher *BC* value indicated that nodes of the GAN were in between many other nodes. Also, a higher *BRC* value suggested that nodes of GAN can reach a higher portion of nodes, which is termed ‘reachability’ in some literature. In addition, it is noted that the GAN had a negative BROC which means the GAN inclined to form a cluster.

In order to further our understanding of the GAN network property, we sorted the eight local parameters’ values in descending order, and counted the number of genes ranked in the top 10, 15 and 20%. Table 5 summarized the results of the rank statistics of the GAN. As seen in Table 5, there is no gene with an *SV* of five ranked in the top 10 and 15%. However, there are three/eight genes which have an *SV* of five/four at the top 20/10%, respectively.

**Table 5:** The number of top-ranked genes by using graph topological analysis.

%\score	5	4	3	2	1
10%	0	8	18	38	99
15%	0	19	23	43	81
20%	3	30	25	41	76

As shown in Table 5, at the 10 and 15% thresholds, the highest score is four, while a closer examination revealed that most of the nodes’ group 3 values, i.e. BRC, were ranked beyond the top 20% threshold. This means information flow or node connecting densely connected components was less likely.

For an *SV* of four and 10% threshold, the eight genes are: *AURKA*, *CYR61*, *DUSP10*, *HSPA1A*, *MED20*, *NFIB*, *NR1D1* and *TCF4*, their roles and related studies were depicted in Table 6. These results provide substantial evidence that top ranked nodes play a role during the stress-inducing process.

**Table 6:** Top-ranked genes, their functional description and supporting study.

Gene	Functional description	Study
*AURKA*	Cell cycle-related genes induced by PDGF-DD in VSMC	Alexander et al. (2012)
*CYR61*	Supports VSMC adhesion and induces VSMC chemotaxis associated with post-angioplasty restenosis	Grzeszkiewicz et al. (2002) and Lee et al. (2007)
*DUSP10*	DUSP10 encodes a dual-specificity MAPK phosphatase that has a principal function in both innate and adaptive immune responses	Weinsheimer et al. (2007)
*HSPA1A*	In VSMC, low-density lipoproteins (LDL) modulate HSP phosphorylation and subcellular localization, affecting action polymerization and cytoskeleton dynamics in rats, increases heart tissue hsp70i expression results in protection of the heart against inadequate blood supply injury	García-Arguinzonis et al. (2010) and Marber et al. (1995)
*MED20*	A repressor of smooth muscle cell differentiation	Beyer et al. (2007)
*NFIB*	A transcriptional inhibitor of p21(CIP), and CDC25A	Dellago et al. (2013)
*NR1D1*	NR1D1 also known as Rev-ErbA, upregulates NF-κB-responsive genes in VSMC	Migita, Morser & Kawai (2004)
*TCF4*	Plays a dual role in vascular remodeling by inhibiting VSMC apoptosis and promoting proliferation	Wang et al. (2002)

Among the eight genes, only *HSPA1A* belongs to the HSP protein family and is also identified as a DEGs, whereas the other seven genes are not DEGs.

If we relaxed the threshold to the 20% level, there is a gene with a *SV* of five, i.e. *IGF1-receptor* (*IGF1R*), which plays an important role in migration, cell cycle progression and survival of VSMC (Cheng & Du, 2007; Li et al., 2008; Okura et al., 2001; Qiu et al., 2014b).

Thus, the above results suggested a useful scenario to identify stress-induced genes by using time series data, and elaborate the network structure by using graphical and cluster analysis. Also, the findings support our suggestion that if the genes have higher degree, betweenness, centrality and densely connected, they may play important roles in VSMC under mechanical stress.

### The results of cluster analysis

For a given *k*, CFinder may return more than one cluster; therefore, we selected the largest *k*-community for cluster analysis. Table 7 summarized the results of the sizes and numbers of clusters for the *k*-communities identified by CFinder. For example, CFinder identified two largest clusters with a size of 56 for the 6-communities.

**Table 7:** Sizes and number of clusters for the *k*-communities identified by CFinder.

*k*	Size (number of clusters)
3	204, 3
4	176, 5, 4
5	136, 7, 5
6	56(2), 11, 7(2), 6(5)
7	43, 42, 9
8	16(2), 12(2), 10, 9, 8
9	14, 11, 10(3)
10	11, 10

**Note:**

The parenthesis denotes the number of clusters identified by CFinder, else only one cluster was found.

Enriched biological pathways’ annotations of communities were given by implementing DAVID. According to the REACTOME (Croft et al., 2011) and KEGG (Kanehisa et al., 2004), pathways with their *p_DAVID_*-values less than 0.05 and ranked among the top ten were reported.

Table 8 summarized the results of the pathway (*p_DAVID_*-value less than 0.05) or disease information and references for the *k*-communities with *k* equals to 7–10. As we noted from Table 8, cluster analysis allowed us to infer more VSMC-related genes (denoted by bold-faced letters) that were not identified by differential expression or topological analysis. We performed the gene set enrichment analysis for communities with higher connectivity only, the same analysis can be applied for *k* equals to 3–6 if necessary.

**Table 8:** Enriched pathway or disease returned by DAVID.

*k*	Gene name	Pathway or disease
10	*DUSP10*	MAPK (Mascall et al., 2012)
	***SH2D4A***	Angiogenesis, VEGF (Mascall et al., 2012; Qiu et al., 2014b)
8, 9	***BCL2L1***	Ras (Lan et al., 2010)
	*CTGF*	Cardiovascular disease
	*HSPA1A*	Restenosis
	***PLAU***	Blood coagulation
7	***BCL2L1***	Ras (Lan et al., 2010; Mascall et al., 2012)
	***BDKRB1***	Cardiovascular disease
	***CCL2***	Cardiovascular disease
	*CTGF*	Cardiovascular disease
	***CDKN1B***	Cardiovascular disease
	***DDB2***	p53 (Dardik et al., 2005; Wójtowicz et al., 2010)
	***DLC1***	PDGF (Hurley et al., 2010; Smyth, 2005)
	***FAM13A***	Angiogenesis
	*HSPA1A*	Restenosis
	*IGF1R*	IGF1R (Arcaro & Guerreiro, 2007; Huang et al., 2014; Jacob et al., 2005; Sebolt-Leopold & Herrera, 2004)
	*MED20*	Gene expression (Boehm & Nabel, 2001)
	*NFIB*	Cellular process (Qiu et al., 2014a)
	*NR1D1*	NFkB (Qiu et al., 2014a)
	***NR4A1***	MAPK (Hinze et al., 2013)
	***PMAIP1***	p53 (Dardik et al., 2005; Wójtowicz et al., 2010)
	***PLAU***	Blood coagulation
	*TCF4*	Cellular process (Fitzgerald et al., 2008)

**Note:**

Bold-faced gene name denote new putative genes identified by cluster analysis; they were not found by differential expression or topological analysis.

The present approach suggested that a combination of the DEGs, topological and cluster analyses may be more beneficial in terms of identifying VSMC-related genes, i.e. narrowed down to a total of 43 (not for a specific *k*-community). In Table 2, there are 23 DEGs obtained from literature. Through topological analysis of the GGM result (with a SV of four and listed in the top 10%) there are eight genes found (listed in Table 6, but one gene is the same in Table 2), so seven genes were counted. Using the same network from GGM analysis, through the Cfinder calculation and followed by enrichment analysis (DAVID), 13 more genes are found to be relevant to VSMC based on the pathway or disease annotations given by DAVID. Therefore, when we added up 23, 7 and 13, 43 DEGs were found.

A possible explanation for this lies in the fact that different methods of analysis obtained different aspects of the microarray data. For instance, the DEG analysis is based on the fold changes of gene expression, the GGM is rested on partial correlation calculation, whereas the cluster analysis made of PPI and biological functional analysis.

### The results of drug repositioning for VSMC proliferation associated diseases

Using a *p_EBAYES_*-value < 0.01, 473 DEGs were identified. The use of these genes to query against cMAP did not return any potential drugs. Therefore, we used a less stringent *p*-value to estimate the association of the DEGs with drugs. A total of 208 and 394 up- and down-regulated DEGs respectively (*p_EBAYES_*-value < 0.05 and cMap enrichment score < 0, which is shown in Section 2 of the Supplemental Information), with at least two consecutive up- and down-regulated time points, were submitted to cMap, where 30 drugs were returned as shown in Table 9. Among these drugs, three of them, i.e. thioridazine, niclosamide, and pyrvinium have in-house experimental determined IC50 activities.

**Table 9:** The potential drugs returned by cMap under *p_cMAP_*-value < 0.05 and cMap enrichment score < 0.

5224221	5707885	Alimemazine	Arachidonic acid	Ascorbic acid
Benzamil	Bromopride	Bucladesine	Calmidazolium	Cicloheximide
Clomifene	Fluspirilene	Gossypol	LM-1685	Loperamide
Lovastatin	LY-294002	Maprotiline	Methylbenzethonium chloride	Niclosamide
Pentamidine	Pimozide	Puromycin	Pyrvinium	Rottlerin
Syrosingopine	Thioridazine	Trolox C	Valinomycin	Wortmannin

For thioridazine, its association with CVD is its deadly adverse effect (Eder et al., 2014), namely, torsades des pointes arrhythmia, a very severe type of irregular heart beating. One research proposed that there was a significant dose–response relationship for increasing heart rate and increasing drug-induced long Q-T syndrome but not other cardiovascular changes (Strachan, Kelly & Bateman, 2004). Although it took a relatively high dose of thioridazine to cause adverse cardiovascular effects, thioridazine is less likely to be a potential drug for VSMC proliferation associated diseases.

Niclosamide has been found to inhibit rapidly mTORC1 signaling which is required for embryonic cardiovascular development and for postnatal maintenance of cardiac structure and function. Also, mTORC1 is necessary for cardiac adaptation to pressure overload and development of compensatory hypertrophy (Balgi et al., 2009; Sciarretta, Volpe & Sadoshima, 2014). Therefore, it may be less possible to be beneficial for VSMC proliferation associated diseases. Another study suggested niclosamide and L-4F co-administered orally in a mouse model would reduce the atherosclerotic lesion, but not niclosamide alone. Thus, niclosamide may interact with L-4F to protect the effective peptide from trypsin digestion; therefore, allowing its absorption (Navab et al., 2009).

For pyrvinium, multiple ex vivo experiments have suggested it can heal a scarring heart (Murakoshi et al., 2013; Saraswati et al., 2010). Moreover, pyrvinium reduced adverse cardiac remodeling (Saraswati et al., 2010).

By use of the NCBI PubChem database, we found that among the 442 DEGs (after excluding genes which do not have identifiable GeneID) there are 31 DEGs, targeted by 73 drugs. Among the 31 DEGs, seven of them, i.e. *CCL*, *CDK*, *CDKN1B*, *IGF1R*, *MMP*, *PLAU* and *TP53*, matched with our 43 DEGs list, i.e. 16.3%.

Among these six families of genes (*CDK* and *CDKN1B* belong to the same family), the IC_db_50 information for five of them, except *IGF1R*, are given in Table 10. We noted that all the drugs in Table 10 had IC_db_50 values, in particular, the IC_db_50 records are available for two families of DEGs, i.e. the CDK and MMP families. Given the fact that the NCBI PubChem resource is not designed specifically for recording CVD drugs; hence, this may limit the number of matching events. For the moment, the findings listed in Table 10 should be important in VSMC proliferation associated disease treatments.

**Table 10:** The IC_db_50 information for *CCL*, *CDK*, *MMP*, *PLAU* and *TP53*.

Drug name	DEG	IC_db_50 (μM)	Study
Acetylsalicylic acid	*TP53*		Redondo et al. (2003)
Amiloride	*PLAU*		Chan et al. (2011) and Liu et al. (2014)
AT7519	*CDK family*	3 IC_db_50 records were found	Wyatt et al. (2008)
Danazol	*CCL3*	–	
Flavopiridol	*CDK family*	31 IC_db_50 records were found	Andres (2004), Dzau, Braun-Dullaeus & Sedding (2002) and Ruef et al. (1999)
Marimastat	*MMP family*	28 IC_db_50 records were found	Peterson et al. (2000)

**Note:**

‘-’ denotes not available.

In summary, given the 30 potential drugs, we had identified the drug targets which are also the DEGs input in cMAP. With the drug target’s IC_db_50 also found in NCBI, the results imply “very” specific drug action mechanism.

Acetylsalicylic acid (ASA) is under clinical trials (clinical trial NCT00501059) for cardiovascular disease treatment and has been demonstrated to inhibit VSMC proliferation (Redondo et al., 2003).

For amiloride, this drug may reduce the increased VMSC hypertrophy by mediating ion transport mechanism which is induced by angiotensin II. Besides, it has been shown that pre-treatment with 5-N,N-hexamethylene amiloride attenuates angiotensin II-induced VSMC proliferation (Liu et al., 2014). Also, the amiloride derivative phenamil attenuates pulmonary vascular remodeling by activating NFAT and the Bone Morphogenetic Protein signaling pathway (Chan et al., 2011). Amiloride is also under clinical trials (clinical trial NCT01195805) for cardiovascular disease treatment.

AT7519, a novel small-molecule multi-cyclin dependent kinase inhibitor, has been evaluated in clinical trials for the treatment of cancers (Dolman et al., 2015; Wyatt et al., 2008).

For AT7519 and flavopiridol, the corresponding clinical trials (clinical trials NCT01183949 and NCT00112723) for myeloma are ongoing. Furthermore, for flavopiridol, it has been reported that it inhibits VSMC growth in vitro and in vivo. Its oral availability and selectivity for CDKs make it a potential therapeutic tool in the treatment of vascular lesions (Andres, 2004; Dzau, Braun-Dullaeus & Sedding, 2002; Ruef et al., 1999).

For marimastat, the clinical trial (clinical trial NCT00261391) for vascular anomalies has completed, and it has been reported that marimastat (a selective but non-specific MMP inhibitor) can prevent intimal hyperplasia in a cultured human internal mammary artery (Peterson et al., 2000). Interestingly, it is known that MMP inhibitors (MMPIs) could suppress the effects of MMPs on VSMC proliferation, perhaps it is the most promising clinical application of MMPIs in vascular medicine.

Taken together, with our finding and the literature support, these above-mentioned five drugs might have chances to be potential drugs for CVDs.

### Targeted genes for VSMC proliferation

Drug-target relationships have been used for drug repositioning. For example, Huang et al. (2015) constructed a weighted and integrated drug-target interactome (WinDTome) in drug repurposing for schizophrenia. Wu, Wang & Chen (2013) merged disease-gene and drug-target relations for drug repositioning. We are motivated to derive the targeted genes for the potential drugs.

The potential drugs in Table 9 were submitted to NCBI to search for their corresponding targeted genes. Finally yielding a total of 38 targeted genes for diseases associated with VSMC proliferation, as shown in Table 11, which are the potential therapeutic targets for future VSMC proliferation diseases clinical trials. For each targeted gene in Table 11, the number in parentheses is the number of associated cMap drugs, and could be regarded as a metric for prioritizing the genes in the list. The *TP53* and *ADRB2* genes ranked top of the lists.

**Table 11:** The genes targeted by the 30 potential drugs.

*NPC1* (20) U	*IL1B* (14) U	*PPP3CA* (10) U	*HSPA1A* (4) U	*PTBP1* (3) U
*CYP51A1* (2) U	*RNASEH1* (1) U	*CDK19* (1) U	*AKT3* (1) U	*INSR* (1) U
*TP53* (23) D	*ADRB2* (23) D	*RGS4* (22) D	*SMPD1* (17) D	*POLI* (17) D
*BAZ2B* (15) D	*MMP14* (15) D	*BCL2L1* (15) D	*NR2F2* (14) D	*SUMO1* (14) D
*VIPR1* (10) D	*EGFR* (10) D	*ID4* (9) D	*RXRA* (7) D	*PLEC* (5) D
*NLRP1* (5) D	*STAT1* (4) D	*WEE1* (4) D	*STK3* (3) D	*SRPK1* (3) D
*SGK1* (3) D	*MKNK2* (3) D	*DUSP1* (2) D	*PLAU* (1) D	*CDC42BPA* (1) D
*CDK2* (1) D	*KDR* (1) D	*SIK1* (1) D		

**Note:**

The parentheses represent the number of associated cMap drugs, U and D represent up and down-regulated respectively.

**Figure 2:**
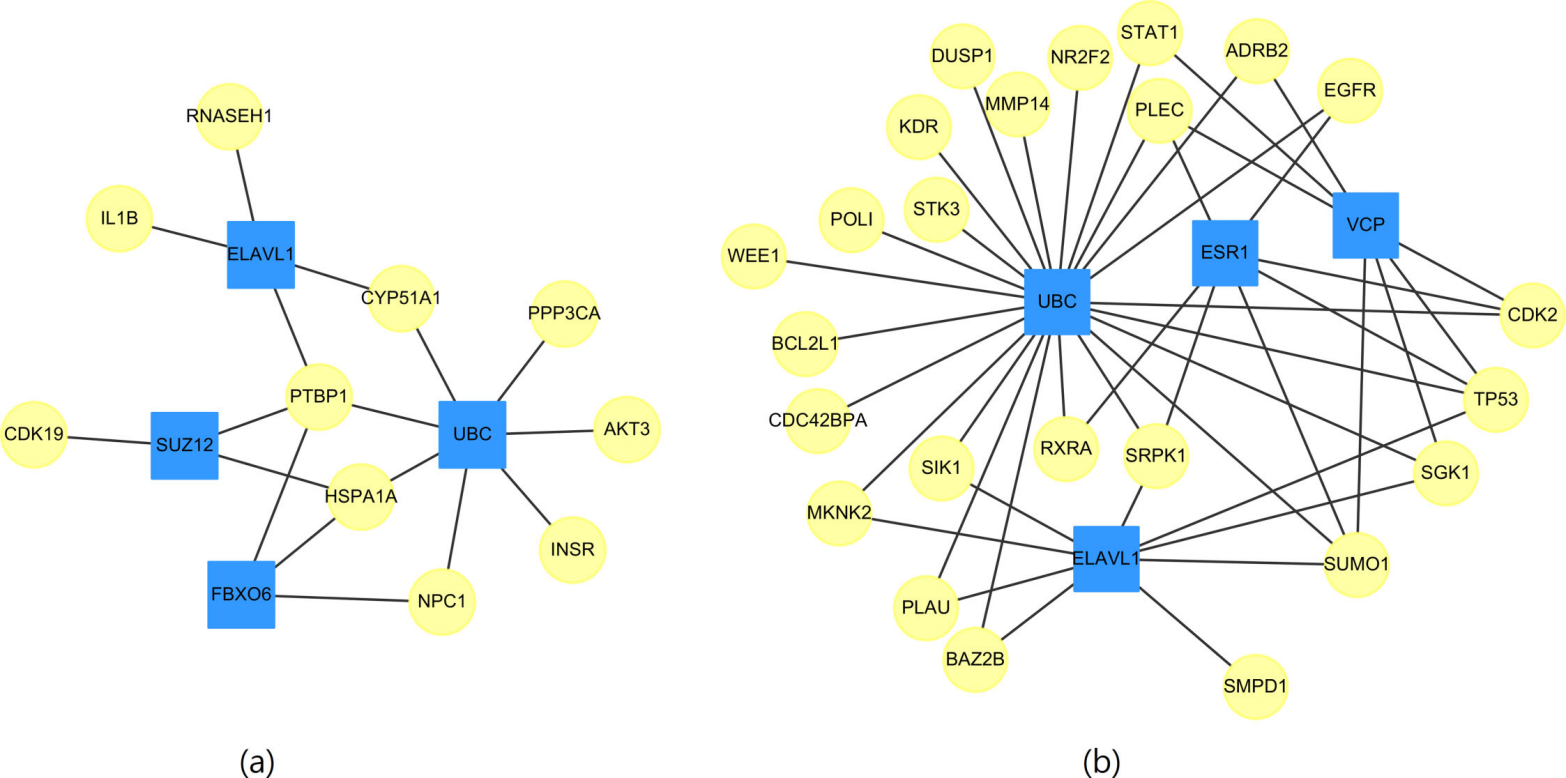
The top four genes (squares) which connect to the largest, the second largest, the third largest and the fourth largest degree of targeted genes (circles) for (A) up-regulated and (B) down-regulated CVDs network.

Whether a particular gene is related to most of the targeted genes in Table 11 is of interest. Therefore, networks of the targeted genes and their adjacent genes in CVDs PPI were constructed. Figure 2 displays the top four genes that exhibit the largest, the second largest, the third largest and the fourth largest degree in CVDs networks. The UBC gene directly interacts with 7(23) targeted genes in up(down) regulated CVDs networks, while the other genes, such as *ELAVL1*, connect to no more than 4(9) targeted genes.

We noted that the ubiquitin-conjugating gene (UBC) is not a DEG. However, as early as 1997, it has been reported by Adam et al. (1997) that UBC is involved in the function or maintenance of the contractile phenotype of VMSC. UBC is responsible for catalyzing the transfer of ubiquitin to substrate proteins is a component of the ubiquitin–proteasome system (*UPS*).

It was known that the *UPS*, regulates the degradation of oxidized, misfolded or damaged proteins, plays a significant role in the control of VSMC phenotype and survival signaling (Demasi & Laurindo, 2012). It has been suggested that the *UPS* may be a therapeutic target in vascular diseases (Willis et al., 2010). Pagan et al. (2013) also postulated that the *UPS* is a promising potential therapeutic target in ischemia-reperfusion injury. We expect the development of novel therapeutic drugs are capable of modulating the *UPS* for CVD treatments.

### Improving drug prediction accuracy by using clustering approach

In the above analysis, a total of 208 and 394 up- and down-regulated DEGs were used to identify 30 potential drugs (as listed in Table 9) for CVDs, and three of them, i.e. thioridazine, niclosamide and pyrvinium have in-house experimental determined IC50 activities. In an attempt to improve the drug prediction accuracy according to IC50 measurements, we further applied graph clustering to extract densely connected clusters of DEGs. A well-known algorithm–ClusterONE (Nepusz, Yu & Paccanaro, 2012) was used for clustering, and the top two largest up- (down-) regulated clusters with sizes 7 and 6 (22 and 8) were selected for analysis (listed in Section 3 of the Supplemental Information). There are four options formed by combining the two up-regulated clusters and the two down-regulated clusters. After submitted to cMap, among the four combinations, we found that by combining the up-regulated cluster of size 6 with the down-regulated cluster with size 22 achieve the highest prediction accuracy according to IC50 measurements, 30.0% (15/50). That is, it predicts 50 drugs, of which 15 drugs were validated as effective by IC50 experiments. Furthermore, combining the up-regulated cluster of size 6 with the down-regulated cluster with size 8 achieve the second highest prediction accuracy according to IC50 measurements, 21.5% (11/51). In contrast, combining the up-regulated cluster of size 7 with the down-regulated cluster with sizes 22 and 8 achieves none and one drug (thapsigargin) is verified by IC50 experiments, respectively.

Out of the 15 predicted potential CVD compounds mentioned in above, 11 of them (73% success rate) have been found to act on the cardiovascular system in the literature. Among them are compounds from various notable class of mechanism including peroxisome-proliferator-activated receptors gamma (PPAR-Ƴ) agonist (15-delta prostaglandin J2), heat shock protein 90 (HSP90) inhibitors (monorden, also known as radicicol; and tanespimycin (17AAG)), histone deacetylase (HDAC) inhibitor (trichostatin A) and anti-fibrotic agent (withaferin a). 15-delta prostaglandin J2 acts as a PPAR agonist which has been shown to elicit protection against myocardial injury through the remote ischemic preconditioning in an in vivo model (Lotz et al., 2011). Mefloquine, an anti-malarial drug, has been demonstrated in multiple studies to possess cardiotoxicity exerting a negative inotropic effect on the heart and certain cardiac arrhythmia (Coker et al., 2000). The HSP90 inhibitor, monorden, was shown to induce heat shock protein expression in neonatal rat cardiomyocytes which ultimately conferred cardioprotection to these cardiomyocytes (Griffin, Valdez & Mestril, 2004). Tanespimycin (17AAG), another HSP90 inhibitor, may also possess similar cardioprotective effects. Parthenolide, isolated from a herb extract, has recently been demonstrated to be able to inhibit the VSMC proliferation by inducing G0/G1 phase cell cycle arrest (Weng et al., 2009). Phenoxybenzamine has been used in open heart surgery, coronary artery bypass grafting (CABG) with a radial artery, and in one study, treatment of the radial artery grafts with phenoxybenzamine was associated with a reduction in perioperative myocardial injury (Kulik et al., 2007). Piperlongumine is an alkaloid extracted from the long pepper, Piper Longum L., which was found to have anti-atherosclerotic action suppressing atherosclerosis plaque formation in vivo (Son et al., 2012). Trichostatin A, an HDAC inhibitor, has been shown to be able to confer cardioprotection against myocardial ischemia/reperfusion injury in vivo in one animal study (Yu et al., 2012). Trifluoperazine is a calmodulin inhibitor which ranks high in the order of effectiveness amongst its same class compounds in protecting the calcium-overloaded myocardium (Hong et al., 2014). Zhao et al. (2012) has reported the use of vorinostat in pulmonary arterial hypertension. Finally in the list is withaferin A, an anti-fibrotic compound against diseases such as cardiac interstitial fibrosis (Challa et al., 2012).

**Figure 3:**
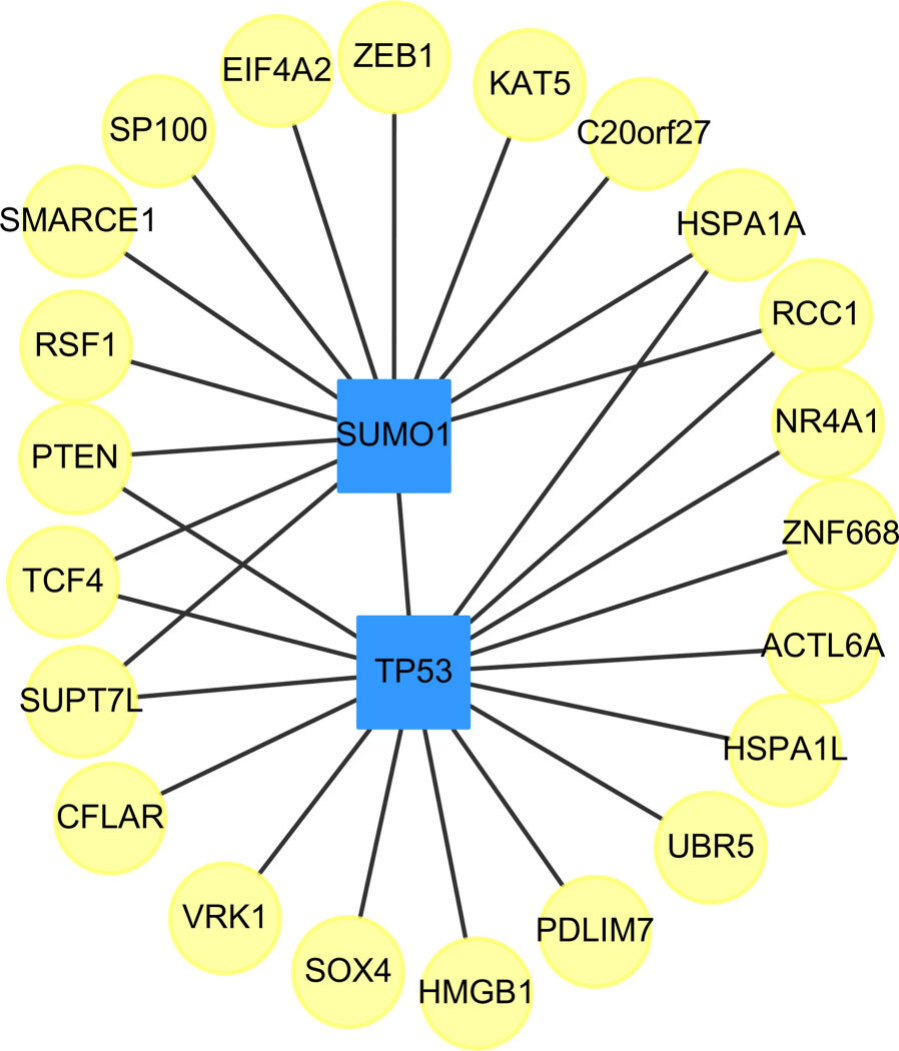
The top two genes (squares) which connect to the largest and the second largest degree of targeted genes (circles) in the down-regulated CVDs PPI after cluster analysis.

We found two drug-targeted genes, TP53, and SUMO1, which are appeared in our prediction with and without the use of the clustering algorithm, ClusterONE. The networks of targeted genes and their adjacent genes in CVDs PPI were shown in Fig. 3. If drugs could indirectly affect down-stream PPI of the TP53 and SUMO1 genes, the therapeutic role of these two genes is worth further exploration.

The small ubiquitin-related modifier (SUMO) family of proteins play an important role in post-translational modification (Mahajan et al., 1997). There are four SUMO genes (SUMO1-4), which encode proteins that are similar to ubiquitin. SUMO plays an important role in PPARgamma Peroxisome proliferator-activated receptor gamma (PPARgamma) activity. PPARgamma is a nuclear receptor regulating inflammation, atherosclerosis, insulin sensitivity and adipogenesis (Lim et al., 2009).

In Table 12, we provided the references for potential cardiovascular-acting mechanisms, which do not have any clinical trial IDs, those are new potential drugs which were not studied in themes of CVD. In other words, our approach is able to identify potential drugs for the treatment of stress-induced CVD. Table 12 lists the drug names, the IC50 values, the drugs’ target genes, i.e. SUMO1 (Gene ID: 7341) and TP53 (Gene ID: 7157), their IC_db_50 values and the supporting studies. In addition, because TP53 is frequently found in liver cancer patients, we further analyzed the IC_liver_50 of these prioritized drugs in three different liver cancer cell lines, including Mahlavu, Huh7, and PLC5 cells. In Section 4 of the Supplemental Information, Table S4 summarized the IC_liver_50 values of those prioritized drugs.

**Table 12:** The MTT and clonogenic assay values (IC50), target gene (DEG) and IC_db_50 information for the cMAP drugs.

Drug name	MTT/Clonogenic IC50 (μM)	DEG	IC_db_50 (μM)	Study*
Lomustine	−/< 10	*SUMO1*	–	–
Parthenolide	> 5/−	*TP53*	15.5	Kurdi & Booz (2007) and Weng et al. (2009)
Phenoxybenzamine	> 5/−	*SUMO1*	–	Corvera et al. (2003) and Kulik et al. (2007)
Piperlongumine	> 5/−	*TP53*	3.16	Son et al. (2012)
		*SUMO1*	–	
Securinine	> 5/−	*TP53*	29.8	–
Sulconazole	−/< 10	*SUMO1*	–	–
Tanespimycin	< 1/−	*SUMO1*	–	Kim et al. (2014)
Thiostrepton	< 5/−	*TP53*	7.38	–
Trifluoperazine	< 10/−	*TP53*	19.95	Hong et al. (2014)
		*SUMO1*	–	
Vorinostat	< 1/−	*TP53*	0.708	Zhao et al. (2012)
		*SUMO1*	–	

**Note:**

* Denotes studies shown evidence related to CVDs.

‘-’ denotes not determined.

In total, there are ten common drugs inferred by both the highest and the second highest predictions. Four drugs are specific to the highest prediction, and one drug is specific to the second highest prediction.

For the rest of the 35 drugs (without IC50), only 21 drugs have identifiable targeted DEGs, in which 11 drugs, i.e. 52%, have potential cardiovascular-acting mechanisms, where seven of them have the IC_db_50 values (see Section 5 of the Supplemental Information).

## Discussion

Abnormal expression of VSMC is a major cause of CVDs. To examine how VSMC react in response to mechanical stress, a three-phase study was proposed to examine this problem by employing time-course microarray data. First, the DEGs were identified by using the moderated t-statistics test package, EBayes. Second, the GAN for VSMC was inferred by using GGM. Finally, graph theory and cluster analysis were employed to predict the last batch of DEGs by analyzing the GAN.

A total of 23 genes are found relevant to VSMC phenotypic modification, which is summarized in Table 2. Those genes are differential expressed. Then, by constructing the GAN, one obtained the dependence among the DEGs, in which this approach allows us to infer more DEGs based on their topological properties of the interaction network. To further explore stress-induced DEGs, we made use of the assumption that highly interact DEGs are assumed playing an important role in VSMC phenotype changes, in which dense interaction regions were identified by using the clustering approach.

Our research has suggested that nodes with top-ranked local topological values and densely interacting regions (modulus) represent stress-induced genes in VSMC, where the results were well supported by the literature. The three phases, complement each other well, each phase emphasizes a different aspect of the GAN.

To identify the potential drugs for vascular diseases involve VSMC proliferation, we made use of the drug-gene interaction databases, cMap. Use of up- and down-regulated DEGs query predict 30 drugs, where three of them have in-house IC50 activities. In other words, the success of the predictions was determined using in-vitro data, i.e. MTT and clonogenic assay. We note that more IC50 hits are expected.

The overall drug prediction can be improved if one applies cluster analysis for the input gene sets before making a query against the cMap database. We performed a gene set enrichment analysis on the input sets, and found that the up-regulated cluster of size 6 and down-regulated clusters of size 22 and 8 are enriched in apoptosis and cellular metabolism processes, where the up-regulated cluster of size 7 is enriched in both non-coding RNA and RNA metabolic process. In other words, using up- and down- regulated gene sets involve with similar BPs result in identifying more IC50 hits; hence, provide more potential therapeutic drugs for treating VSMC related diseases.

As we stated in the ‘Drug repositioning’ section, both cancers and the VSMC proliferation process involved the same signal transduction pathways; therefore, it is hypotheized that cancer drug molecules may be repositioned for treating VSMC-associated CVDs. Table 12 summarized the IC50 (lung cancer cells) activities for the ten prioritized drugs. We further analyzed the IC_liver_50 of the prioritized drugs in three different liver cancer cell lines and found that eight drugs shown IC50 assay activity (Table S5). Although the present study has yielded promising findings, we note that further experiments should be pursued for VSMC before the hypothesis is accepted.

## Conclusions

In conclusion, biological networks are composed of functional related modules, which play an essential role in many BPs. This research proposed a computational framework; i.e. use of GGM, multiple topological parameters and cluster analysis, to dissect network structures, which may shed light on the mechanism of CVDs. Furthermore, potential drugs and their targeted genes were identified from cMap and NCBI PubChem, and certain potential drugs have been tested for effectiveness by in vitro anti-tumor effects and clinical trials. Interestingly, the UBC gene dominates most of the targeted genes associated with CVDs network, so its role in the cancer pathway warrants further investigation. Finally, by applying cluster algorithm to the GAN, we achieved a substantial increase in the number of predicted potential drugs for CVDs according to the IC50 measurements. Consequently, we have been able to assess the potential existing drugs to identify novel indications, which may be helpful in drug repositioning discovery for CVDs.

## Supplemental Information

10.7717/peerj.2478/supp-1 Supplemental Information 1 Supplemental Materials.

10.7717/peerj.2478/supp-2 Supplemental Information 2 File S1. Gene expression profiles of the VSMC in response to a cyclical mechanical strain at time t = 0 h.

10.7717/peerj.2478/supp-3 Supplemental Information 3 File S2. Gene expression profiles of the VSMC in response to a cyclical mechanical strain at time t = 0 h (Replicate).

10.7717/peerj.2478/supp-4 Supplemental Information 4 File S3. Gene expression profiles of the VSMC in response to a cyclical mechanical strain at time t = 2 h.

10.7717/peerj.2478/supp-5 Supplemental Information 5 File S4. Gene expression profiles of the VSMC in response to a cyclical mechanical strain at time t = 0 h (Replicate).

10.7717/peerj.2478/supp-6 Supplemental Information 6 File S5. Gene expression profiles of the VSMC in response to a cyclical mechanical strain at time t = 4 h.

10.7717/peerj.2478/supp-7 Supplemental Information 7 File S4. Gene expression profiles of the VSMC in response to a cyclical mechanical strain at time t = 4 h (Replicate).

10.7717/peerj.2478/supp-8 Supplemental Information 8 File S7. Gene expression profiles of the VSMC in response to a cyclical mechanical strain at time t = 24 h.

10.7717/peerj.2478/supp-9 Supplemental Information 9 File S8. Gene expression profiles of the VSMC in response to a cyclical mechanical strain at time t = 24 h (Replicate).

10.7717/peerj.2478/supp-10 Supplemental Information 10 A list of up-regulated DEGs.

10.7717/peerj.2478/supp-11 Supplemental Information 11 A list of down-regulated DEGs.

10.7717/peerj.2478/supp-12 Supplemental Information 12 The top two largest up- (down-) regulated clusters with sizes 7 and 6 (22 and 8) after ClusterONE analysis.

10.7717/peerj.2478/supp-13 Supplemental Information 13 The results of IC_liver_50 values for three liver cancer cell lines, including Mahlavu, Huh7, and PLC5 cells. The table summarized the IC_liver_50 values after the treatment of 72 h.

10.7717/peerj.2478/supp-14 Supplemental Information 14 The results of cMAP drugs, their target genes (DEG), the IC_db_50 values, and potential (cardio)vascular-acting mechanisms on CVDs information.

## Additional Information and Declarations

### Data Deposition

The following information was supplied regarding data availability: The research in this article did not generate any raw data.
