# Supplementary material for: Identify potential drugs for cardiovascular diseases caused by stress-induced genes in vascular smooth muscle cells
Source: PeerJ. 2016 Sep 28;4:e2478. doi: 10.7717/peerj.2478 (PMC5045879; doi:10.7717/peerj.2478)
Supplement: Supplemental Information 1 [file peerj-04-2478-s001.docx]

# Supplementary material: Identify Potential Drugs for Cardiovascular Diseases Derived from Stress-induced Genes in Vascular Smooth Muscle Cells

Chien-Hung Huang^1^, Jin-Shuei Ciou^2^, Shun-Tsung Chen^2^, Victor C. Kok^2,3^, Yi Chung^2^, Chi-Ying F. Huang^5^, Ke-Rung Tzeng^2^, Jeffrey J. P. Tsai^2^, Nilubon Kurubanjerdjit^4^, Ka-Lok Ng^2,6^

^1^Department of Computer Science and Information Engineering, National Formosa University, Yun-Lin, Taiwan 63205

^2^Department of Bioinformatics and Medical Engineering, Asia University, Taichung, Taiwan 41354

^3^Division of Medical Oncology, Kuang Tien General Hospital Cancer Center, Taichung, Taiwan 43303

^4^School of Information Technology, Mae Fah Luang University, Chiang Rai, Thailand 57100

^5^Institute of Biopharmaceutical Sciences, National Yang-Ming University, Taipei, Taiwan 112

^6^Department of Medical Research, China Medical University Hospital, China Medical University, Taichung 40402, Taiwan

Corresponding Author: [ppiddi@gmail.com](mailto:ppiddi@gmail.com)

## **1. Topological Graph Theory**

Average Graph Distance (AGD)

In a biological network, the distance *d*(*i*, *j*) between two nodes *i* and *j* is just the length of a shortest path joining *i* and *j* (if it exists). The average graph distance is the average over all distances *d*(*i*, *j*) of node pairs *i* and *j* in the network.

Diameter

Diameter in a network is defined as the largest number of vertices, which must be traversed in order to travel from one node to another.

Network Efficiency (NE)

Network efficiency is a global measure of network integrity related to the shortest path length among each pair of elements within the network. The network efficiency is expressed as the sum of the reciprocals of the shortest path lengths between all pairs of elements of a node *i* and is defined as follows (Csermely et al. 2005):

|  |  | (1) |
| --- | --- | --- |

where the distance *d*(*i*, *j*) denotes the shortest path from node *i* to node *j*, and *N* denotes the total number of nodes in the network.

Degree Centrality (DC)

Degree centrality shows that an important node is involved in a large number of interactions. Degree centrality of a node *i*, *DC*(*i*), denotes the node degree of node *i*. The *DC* of a node *i* in a network is defined by:

|  | $DC(i)=\frac{\sum A_{ij}}{N-1}$ | (2) |
| --- | --- | --- |

where A*_ij_* is the corresponding entry value in adjacency matrix A and N denotes the total number of nodes in the network.

Betweenness Centrality (BC)

Betweenness centrality is the accumulation fraction of all shortest paths passing on a given vertex. The betweenness centrality *BC*(*i*) of a node *i* is computed as follows:

|  |  | (3) |
| --- | --- | --- |

where *s* and *t* are nodes in the network different from *i*, σ*_st_* denotes the number of shortest paths from *s* to *t*, and σ*_st_*(*i*) is the number of shortest paths from *s* to *t* that pass through *i*.

The betweenness centrality value for each node *i* is normalized by dividing by the number of node pairs excluding *i*: (*N*-1)(*N*-2)/2, where *N* is the total number of nodes in the connected component that *i* belongs to. The betweenness centrality of a node reflects the amount of control that this node employs over the interactions of other nodes in the network (Pavlopoulos et al. 2011).

Bridging Centrality (BRC)

Many centrality properties concentrate on central components and overlook another essential topological aspect in networks. The bridging centrality is a node centrality index based on information flow and topological locality in networks. A bridging node is a node connecting densely connected components in a graph. It is the product of the [betweenness centrality](http://www.cbmc.it/fastcent/doc/Betweenness.htm) *BC* and the bridging coefficient *BCO*, which measures the global and local features of a node respectively. The bridging coefficient of a node determines the extent of how well the node is located between high degree nodes. The bridging coefficient of a node *i* is defined by (Hwang. et al. In KDD'06 August 20-23. Philadelphia, PA, USA; 2006. ):

|  |  | (4) |
| --- | --- | --- |

where *d*(*i*) is the degree of node *i*, and *N*(*i*) is the set of neighbors of node *i*.

Specifically, the bridging centrality BRC(*i*) of node *i* is defined by (Hwang. et al. In KDD'06 August 20-23. Philadelphia, PA, USA; 2006. ):

|  |  | (5) |
| --- | --- | --- |

The bridging centrality can identify bridging nodes, which are the nodes with more information passing through them.

Closeness Centrality (CC)

The adjacency matrix *A* of a graph can be expressed in terms of its eigenvalues and eigenvectors as

|  |  | (6) |
| --- | --- | --- |

whereis the eigenvalue matrix and *x_i_* is the eigenvector. Since *A* is a symmetric matrix, all of its eigenvectors are orthogonal.

The closeness centrality (CC) of node *i* is the sum of the shortest path lengths over all pairs of nodes in the network. The CC of a node *i* in a network is defined by:

(7)

where the distance *d*(*i*, *j*) denotes the shortest path from node *i* to node *j*, and *N* denotes the total number of nodes in the network.

Closeness centrality indicates important nodes that can communicate quickly with other nodes of the network. Closeness centrality has been used to identify the top central metabolites in metabolic networks (Ma & Zeng 2003) and it has been chosen as the best centrality measure that can be used to extract the metabolic core of a network (Rosa. et al. 2008).

Eccentricity Centrality (EC)

Eccentricity centrality (EC) is the longest distance required for a given node to reach the entire network. Let *dist*_max_(*i*) denote the maximal distance node *i* to any other node *j*. The *EC* of a node *i* in a network is defined by:

|  |  | (8) |
| --- | --- | --- |

In biological networks, bioentities with high eccentricity are easily functionally reachable by other components of the network, and thus can readily perceive neighboring changes (Chavali et al. 2010).

Clustering Coefficient (CLC)

The clustering coefficient is the measurement that shows the tendency of a graph to be divided into clusters. Clustering coefficient lies between zero and one that is zero when there is no clustering, and one for the network consisting of disjoint cliques. The clustering coefficient is the ratio *e_i_*/*M*, where *e_i_* is the number of edges between the neighbors of *i,* and *M* is the maximum number of edges that could possibly exist between the neighbors of *i*.

The clustering coefficient of a node *i* is defined as (Soffer & Vazquez 2005):

|  |  | (9) |
| --- | --- | --- |

where *N*(*i*) is the set of neighbors of node *i*, is the number of neighbors of *i* and *e_i_* is the number of connected pairs between all neighbors of *i*.

Brokering Coefficient (BROC)

The Brokering coefficient is defined by (Cai et al. 2010):

|  |  | (10) |
| --- | --- | --- |

where *DC*(*i*) is the degree of node *i* and *CLC*(*i*) is the clustering coefficient of a node *i*. Since the BROC value is the difference (logarithmic) between node degree and the cluster coefficient; therefore, a negative BROC value indicates that the network inclines to form a cluster.

Local Average Connectivity (LAC)

Let *N_i_* be the set of neighbors of node *i*, and the subgraph induced by *N_i_* be *C_i_*. For each node *j* in *C_i_*, its local connectivity in *C_i_* is represented as , which represents the number of other nodes in *C_i_* that it connects directly with. Formally, the local average connectivity of a node *i* is defined as follows (Li et al. 2011):

|  |  | (11) |
| --- | --- | --- |

LAC can determine a node’s essentiality by evaluating the relationship between a node and its neighbors.

# References

Cai JJ, Borenstein E, and Petrov DA. 2010. Broker genes in human disease. *Genome Biol Evol* 2:815-825. 10.1093/gbe/evq064

Chavali S, Barrenas F, Kanduri K, and Benson M. 2010. Network properties of human disease genes with pleiotropic effects. *BMC Syst Biol* 4:78. 10.1186/1752-0509-4-78

Csermely P, Agoston V, and Pongor S. 2005. The efficiency of multi-target drugs: the network approach might help drug design. *Trends Pharmacol Sci* 26:178-182. 10.1016/j.tips.2005.02.007

Hwang. WC, Cho. Y-R, Zhang. A, and Ramanathan. M. In KDD'06 August 20-23. Philadelphia, PA, USA; 2006. . Bridging Centrality: Identifying Bridging Nodes in Scale-free Networks.

Li M, Wang J, Chen X, Wang H, and Pan Y. 2011. A local average connectivity-based method for identifying essential proteins from the network level. *Comput Biol Chem* 35:143-150. 10.1016/j.compbiolchem.2011.04.002

Ma HW, and Zeng AP. 2003. The connectivity structure, giant strong component and centrality of metabolic networks. *Bioinformatics* 19:1423-1430.

Pavlopoulos GA, Secrier M, Moschopoulos CN, Soldatos TG, Kossida S, Aerts J, Schneider R, and Bagos PG. 2011. Using graph theory to analyze biological networks. *BioData Min* 4:10. 10.1186/1756-0381-4-10

Rosa. dSMr, Hongwu. M, and An-Ping. Z. 2008. Centrality, Network Capacity, and Modularity as Parameters to Analyze the Core-Periphery Structure in Metabolic Networks. *Proceedings of the IEEE 2008* 96(8):1411 - 1420.

Soffer SN, and Vazquez A. 2005. Network clustering coefficient without degree-correlation biases. *Phys Rev E Stat Nonlin Soft Matter Phys* 71:057101.

## **2. A list of up- and down-regulated DEGs**

Table S1. A list of up-regulated DEGs.

| *ADGRL4* | *AHNAK* | *AKT3* | *AMD1* | *AOC4P* | *ARHGEF12* | *ARMCX2* |
| --- | --- | --- | --- | --- | --- | --- |
| *ARMCX6* | *ASAH1* | *ASPH* | *ATP6V0E1* | *AZIN1* | *BAG3* | *BIN3* |
| *BTG3* | *C7orf55-LUC7L2* | *CACNA1F* | *CCDC51* | *CCL2* | *CCL20* | *CD163* |
| *CD164* | *CDH15* | *CDH2* | *CDK19* | *CDKN2A* | *CEACAM1* | *CERS4* |
| *CHD3* | *CHODL* | *CKLF* | *CNTNAP2* | *CREBL2* | *CRY1* | *CTNND1* |
| *CXorf40B* | *CYB5R4* | *CYP51A1* | *DAB2* | *DICER1* | *DKC1* | *DNAJA1* |
| *DNMBP* | *DOHH* | *DPY19L4* | *DRAM1* | *DSCR4* | *DVL3* | *EHD1* |
| *EIF4A2* | *ELAVL3* | *ENTPD7* | *EPO* | *ESPL1* | *ETNK2* | *FABP2* |
| *FAM57A* | *FAS* | *FGF2* | *FNDC4* | *FOXA2* | *FST* | *GABRA4* |
| *GEMIN4* | *GM2A* | *GNL3* | *GNPAT* | *GOLPH3L* | *GPR161* | *GPRC5A* |
| *GRAP* | *GRPEL1* | *GRWD1* | *HADH* | *HAPLN2* | *HIST1H2BC* | *HIST1H2BE* |
| *HIST1H2BF* | *HIST1H2BG* | *HIST1H2BI* | *HMGCS1* | *HRH4* | *HS2ST1* | *HSPA1A* |
| *HSPA1B* | *HSPA1L* | *HSPB7* | *HSPH1* | *IDH3B* | *IGHG1* | *IL1B* |
| *IL7R* | *INO80B* | *INSR* | *KIAA0226L* | *KIAA0907* | *KIF15* | *KPNA2* |
| *LMAN2L* | *LMBRD1* | *LOC100129518* | *LOC100996724* | *LOC100996761* | *LOC100996792* | *LOC101928189* |
| *LPCAT4* | *LRRD1* | *LUC7L2* | *LUC7L3* | *LYPLA2* | *MAP2K3* | *MAP3K4* |
| *MAPRE2* | *MAT2A* | *MATN1* | *MCTP2* | *ME1* | *MEGF9* | *MIR1248* |
| *MIR1292* | *MIR4745* | *MIR664B* | *MLF2* | *MMD* | *MMP10* | *MRPL52* |
| *MTRR* | *MYL10* | *NCALD* | *NCAPH* | *NEFM* | *NEU1* | *NFATC2IP* |
| *NGF* | *NOLC1* | *NOP2* | *NOP56* | *NPC1* | *NPPC* | *NT5DC3* |
| *NUPL1* | *OAS2* | *OR10H3* | *PDE4DIP* | *PDGFB* | *PDLIM4* | *PDXK* |
| *PIGO* | *PKD2* | *PMCH* | *PNPLA2* | *PPAT* | *PPIF* | *PPP3CA* |
| *PRPF3* | *PRPF4* | *PSPC1* | *PTBP1* | *PTCRA* | *RBM25* | *RBPMS* |
| *RCBTB2* | *REG3A* | *RFC5* | *RNASEH1* | *RRS1* | *RSRP1* | *S100A5* |
| *SATB1* | *SDAD1* | *SDCBP* | *SGCE* | *SKP2* | *SLC22A7* | *SLC25A22* |
| *SLC39A8* | *SLC5A3* | *SMARCA5* | *SMOX* | *SMTN* | *SNORA4* | *SNORA56* |
| *SNORA63* | *SNORA81* | *SNORD110* | *SNORD19B* | *SNORD2* | *SNORD57* | *SNORD86* |
| *SOCS2* | *SOD2* | *SON* | *SQSTM1* | *SRR* | *SSX3* | *ST3GAL1* |
| *ST8SIA4* | *STAT1* | *STAT2* | *STATH* | *TBX5* | *TCL6* | *THOC1* |
| *TM4SF1* | *TMEM14A* | *TMX2-CTNND1* | *TNPO3* | *TPP1* | *TRIM27* | *TRIM31* |
| *TRIM32* | *TTC12* | *TUBGCP4* | *TULP2* | *TUSC3* | *WSB2* | *ZBTB11* |
| *ZDHHC18* | *ZNF189* | *ZNF318* | *ZNF467* | *ZSCAN32* | *ZYX* |  |

Table S2. A list of down-regulated DEGs.

| *2-Mar* | *7-Mar* | *ABCA1* | *ACTL6A* | *ADGRG1* | *ADRB2* | *AGL* |
| --- | --- | --- | --- | --- | --- | --- |
| *AHNAK2* | *AKAP13* | *AKAP9* | *AMIGO2* | *AMOTL2* | *ANKRD11* | *ARHGAP35* |
| *ARHGDIA* | *ARL4A* | *ARMC9* | *ASF1A* | *ASS1* | *ATAD2* | *ATF5* |
| *ATP5D* | *B2M* | *BAX* | *BAZ2B* | *BBX* | *BCL2L1* | *BCL6* |
| *BHLHE40* | *BLVRA* | *BMPR1A* | *C10orf76* | *C14orf1* | *C20orf27* | *CALHM2* |
| *CALM1* | *CALM2* | *CALM3* | *CAMSAP2* | *CAV2* | *CCDC91* | *CCNG2* |
| *CCNL1* | *CCP110* | *CCPG1* | *CDC42BPA* | *CDC42EP3* | *CDCP1* | *CDH13* |
| *CDK2* | *CDK5RAP3* | *CDS2* | *CEP170* | *CEP170P1* | *CEP350* | *CEP55* |
| *CEP57* | *CEP76* | *CFLAR* | *CHAF1B* | *CITED2* | *CLASP1* | *CLCC1* |
| *CLIC4* | *CLN5* | *CNOT2* | *CPQ* | *CRBN* | *CREBL2* | *CRTC3* |
| *CRYZ* | *CTGF* | *CUL5* | *CXCL2* | *CYB5B* | *CYB5R1* | *CYR61* |
| *DAAM1* | *DEK* | *DHX40* | *DIAPH2* | *DKK2* | *DLC1* | *DNASE2* |
| *DPYD* | *DUSP1* | *DYX1C1-CCPG1* | *ECSIT* | *ECT2* | *EGFR* | *EGR2* |
| *EGR3* | *ELP4* | *ENC1* | *ENOSF1* | *EPRS* | *ERMAP* | *ETV5* |
| *EXOC2* | *EXOSC8* | *F2R* | *FAM149B1* | *FAM172A* | *FAM20B* | *FAM21C* |
| *FAM69A* | *FANCG* | *FBXO16* | *FBXW11* | *FCHSD2* | *FEM1B* | *FKBP1B* |
| *FMR1* | *FOSB* | *FPGT* | *FRYL* | *FSCN1* | *GALNT10* | *GANAB* |
| *GCOM1* | *GLTSCR1L* | *GNS* | *GPBP1L1* | *GPD1L* | *GTPBP2* | *GULP1* |
| *H2AFY* | *HBS1L* | *HDGFRP3* | *HECA* | *HECTD4* | *HHEX* | *HHLA3* |
| *HIBCH* | *HIST1H1C* | *HMGB1* | *HMGB3* | *HMGXB4* | *HNRNPD* | *HSPG2* |
| *ID4* | *IFI16* | *IFNGR1* | *IFT74* | *IL1A* | *IL23A* | *IRF9* |
| *JUNB* | *JUP* | *KAT5* | *KAT6B* | *KCNJ2* | *KDR* | *KIAA0355* |
| *KIAA1109* | *KLF4* | *KLF9* | *KLHL22* | *KLHL24* | *KLHL7* | *LARP6* |
| *LGR5* | *LINC00623* | *LINC00869* | *LMO4* | *LRRC1* | *LRRC49* | *LYRM1* |
| *MACF1* | *MAP3K8* | *MARCKS* | *MBNL2* | *MDC1* | *MED14* | *MFAP4* |
| *MGEA5* | *MIR4680* | *MIR6787* | *MKL2* | *MKNK2* | *MKRN1* | *MMP14* |
| *MOCOS* | *MRE11A* | *MSC* | *MTHFD1* | *MTMR3* | *MUM1* | *MYRIP* |
| *NBN* | *NCAPG2* | *NCK2* | *NCOR1* | *NDUFB7* | *NET1* | *NLRP1* |
| *NMI* | *NMRK1* | *NPAS2* | *NPEPPS* | *NR2F1* | *NR2F2* | *NR4A1* |
| *NR4A2* | *NRG1* | *NSL1* | *NT5C* | *NTAN1* | *NTM* | *NUDT9* |
| *NUMA1* | *NUP133* | *NUP155* | *NUPR1* | *OBFC1* | *OIP5* | *PALMD* |
| *PANK4* | *PAPOLA* | *PARP8* | *PAWR* | *PCBP4* | *PCIF1* | *PCMTD2* |
| *PCYOX1* | *PDCD4* | *PDLIM7* | *PDP1* | *PDS5A* | *PFDN4* | *PHACTR4* |
| *PHB* | *PHF21A* | *PHF3* | *PHKB* | *PICALM* | *PLAU* | *PLCL1* |
| *PLEC* | *PLIN2* | *PLSCR1* | *PMAIP1* | *PMS1* | *POLI* | *POLR2M* |
| *POMGNT1* | *PORCN* | *PPAP2B* | *PPM1A* | *PPM1D* | *PPP1CB* | *PPP2R3C* |
| *PPWD1* | *PRKD3* | *PRPSAP2* | *PSD3* | *PSPH* | *PSPHP1* | *PTEN* |
| *PTGER4* | *PTPRF* | *PUM2* | *RAB3GAP1* | *RAB4A* | *RAD21* | *RAD50* |
| *RALBP1* | *RARA* | *RB1CC1* | *RBAK* | *RBFOX2* | *RBPMS* | *RCAN1* |
| *RCBTB1* | *RCC1* | *REV3L* | *RFC1* | *RFC4* | *RFXANK* | *RGL1* |
| *RGS4* | *RHOB* | *RHOBTB3* | *RHOT1* | *RNF126* | *RNF41* | *ROBO1* |
| *RSF1* | *RXRA* | *SARDH* | *SAV1* | *SCAF11* | *SDC2* | *SGCB* |
| *SGK1* | *SHB* | *SIK1* | *SLC16A3* | *SLC17A9* | *SLC1A4* | *SLC25A12* |
| *SLC26A2* | *SLC35A5* | *SMARCE1* | *SMPD1* | *SNAP23* | *SNX13* | *SNX2* |
| *SOCS5* | *SOX4* | *SP100* | *SP110* | *SPHAR* | *SPTBN1* | *SRPK1* |
| *SRSF5* | *STAG2* | *STAT1* | *STAT6* | *STK3* | *STX16* | *SULF1* |
| *SUMO1* | *SUPT7L* | *SUZ12* | *SUZ12P1* | *SYT11* | *TBC1D22A* | *TBC1D2B* |
| *TBX2* | *TCAF1* | *TCF4* | *TCF7L2* | *TES* | *TIA1* | *TMEM123* |
| *TMEM242* | *TMEM35* | *TMEM38B* | *TNFAIP6* | *TP53* | *TP53I11* | *TRAK1* |
| *TRBV19* | *TRIB2* | *TRIB3* | *TRIM2* | *TSPAN13* | *TTLL7* | *UBE2W* |
| *UBR5* | *UGCG* | *USP46* | *UVRAG* | *VCAN* | *VGLL4* | *VIPR1* |
| *VRK1* | *WBP1L* | *WDR11* | *WEE1* | *WIPF1* | *WSB1* | *WWTR1* |
| *XPO1* | *YKT6* | *YWHAE* | *ZBTB20* | *ZEB1* | *ZFAND5* | *ZFP36L1* |
| *ZMYM4* | *ZNF292* | *ZNF32* | *ZNF331* | *ZNF395* | *ZNF668* |  |

**3. The cluster data after ClusterONE analysis**

Table S3. The top two largest up- (down-) regulated clusters with sizes 7 and 6 (22 and 8) after ClusterONE analysis.

| Up-regulated cluster (7 DEGS) | | | | | | | |
| --- | --- | --- | --- | --- | --- | --- | --- |
| *CDKN2A* | *GNL3* | *GPRC5A* | *NOLC1* | *NOP2* | *NOP56* | *RRS1* |  |
| Up-regulated cluster (6 DEGS) | | | | | | | |
| *BAG3* | *DOHH* | *EIF4A2* | *HSPA1A* | *HSPA1L* | *MLF2* |  |  |
| Down-regulated cluster (22 DEGS) | | | | | | | |
| *ACTL6A* | *C20orf27* | *CFLAR* | *HMGB1* | *KAT5* | *KLF4* | *NR4A1* | *PDLIM7* |
| *PTEN* | *RCC1* | *RSF1* | *SMARCE1* | *SOX4* | *SP100* | *SUMO1* | *SUPT7L* |
| *TCF4* | *TP53* | *UBR5* | *VRK1* | *ZEB1* | *ZNF668* |  |  |
| Down-regulated cluster (8 DEGS) | | | | | | | |
| *FBXW11* | *KDR* | *PDCD4* | *PFDN4* | *RCAN1* | *SHB* | *WEE1* | *ZNF395* |

## **4.** **The in-house IC_liver_50 experimental measurement for three liver cancer cell lines**

Table S4. The results of IC_liver_50 values for three liver cancer cell lines, including Mahlavu, Huh7, and PLC5 cells. The table summarized the IC_liver_50 values after the treatment of 72 hours.

| Drug | Annotation | IC_liver_50 values of 72h (μM) | | |
| --- | --- | --- | --- | --- |
|  |  | Mahlavu | HuH7 | PLC5 |
| lomustine | Chemotherapy for brain tumor | - | - | - |
| parthenolide | Potential proteasome inhibitor/ NF-kB/ HSP inhibitor | 1~3.3 | > 10 | - |
| phenoxybenzamine | Alpha-receptor antagonist for hypertension | > 10 | >10 | >10 |
| piperlongumine | Modulator of ROS levels; Potential anti-neoplastics | 0.33~1 | 3.3~10 | 3.3~10 |
| securinine | Selective GABA receptor antagonist | ~10 | >10 | >10 |
| sulconazole | Anti-fungal agent | > 10 | >10 | >10 |
| tanespimycin | HSP90 inhibitor | - | - | - |
| thiostrepton | FOXM1 inhibitor/ Cancer stem cell inhibitor/ Potent HSP inhibitor | 1~3.3 | 3.3~10 | >10 |
| trifluoperazine | Anti-psychotic agent/ Cancer stem cell inhibitor/ Autophagy | >10 | >10 | >10 |
| vorinostat | HDAC inhibitor | 3.3~10 | 1~3.3 | >10 |

‘-‘ denotes not determined

**5.** **The demonstration of targeted VSMC drugs for its efficacy**

Table S5. The results of cMAP drugs, their target genes (DEG), the IC_db_50 values, and potential (cardio)vascular-acting mechanisms on CVDs information.

| **Drug name** | **Target gene (DEG)** | **IC_db_50 (μM)** | **Potential (cardio)vascular-acting mechanisms on CVD** |
| --- | --- | --- | --- |
| ***No study found yet on CVD/VSMC acting mechanisms.*** | | | |
| alpha-ergocryptine | TP53 | - | No study found on CVD/VSMC. |
|  | SUMO1 | - |  |
| carmustine | TP53 | 1.188 | No study found on CVD/VSMC. |
|  | SUMO1 | - |  |
| chlorambucil | HSPA1A | - | No study found on CVD/VSMC. |
|  | TP53 | 22.39 |  |
|  | SUMO1 | - |  |
| cyclacillin | TP53 | - | No study found on CVD/VSMC. |
| dienestrol | HSPA1A |  | No study found on CVD/VSMC. |
|  | TP53 | 3.916 |  |
|  | SUMO1 | - |  |
| flutamide | HSPA1A | - | No study found on CVD/VSMC. |
|  | TP53 | 73.31 |  |
|  | SUMO1 | - |  |
| hexestrol | TP53 | - | No study found on CVD/VSMC. |
|  | SUMO1 | - |  |
| isoxicam | HSPA1A | - | No study found on CVD/VSMC. |
|  | TP53 | - |  |
|  | SUMO1 | - |  |
| rifabutin | TP53 | 59.77 | No study found on CVD/VSMC. |
|  | SUMO1 | - |  |
| semustine | TP53 | - | No study found on CVD/VSMC. |
|  | SUMO1 | - |  |
| ***Potential effects acting on CVD/VSMC with demonstrated mechanisms.*** | | | |
| cimetidine | HSPA1A | - | Results of in vivo and in vitro assays showed that histamine deficiency promotes cardiomyocytes apoptosis. Administering cimetidine demonstrated a protective effect of histamine against myocardial injury (Deng et al. 2015). |
|  | TP53 | 60.57 |  |
|  | SUMO1 | - |  |
| cytisine | TP53 | - | A nicotinic receptor agonist. The cardiovascular effects are due to activation at autonomic ganglia involving nicotinic receptor subtypes other than α4, α7, or β2 (Jutkiewicz et al. 2013). |
|  | SUMO1 | - |  |
| disulfiram | HSPA1A | - | Oral administration of disulfiram, an inhibitor of Cu/Zn superoxide dismutase (SOD), inhibited angiogenesis in Tg-SOD mice as well as in CD1 nude mice (Marikovsky et al. 2002). |
|  | TP53 | 25.12 |  |
|  | SUMO1 | - |  |
| ebselen | HSPA1A | - | Ebselen may be potential treatments for retinopathies that feature oxidative stress-mediated damage to glia and the microvasculature (Tan et al. 2015). |
|  | TP53 | 7.943 |  |
|  | SUMO1 | - |  |
| equilin | HSPA1A | - | Upregulation of SCD gene expression  with equilin treatment may contribute to a possible protective role of this estrogen in  the mesenteric arteries (Mark-Kappeler et al. 2011). |
|  | TP53 | 76.96 |  |
|  | SUMO1 | - |  |
| hecogenin | TP53 | - | Hecogenin acetate is able to modulate reactive species production, inducing cell cycle arrest and senescence and also modulating ERK1/2 phosphorylation and MMP-2 production (Gasparotto et al. 2014). |
| monastrol | TP53 | - | A kinesin Eg5 inhibitor. Kinesins are known to perform mechanical work to power a variety of cellular functions, from mitosis to organelle transport (Yin et al. 2010). |
| mycophenolic acid | TP53 | 0.06766 | Mycophenolate mofetil has anti-atherogenic effects at the level of endothelial cells, monocytes/ macrophages, smooth muscle cells and dendritic cells. It also exhibits anti-oxidative properties (Olejarz et al. 2014). |
|  | SUMO1 | - |  |
| pralidoxime | HSPA1A | - | Cardiac and peripheral vascular effects of pralidoxime chloride (Barnes et al. 1972). |
|  | TP53 | - |  |
|  | SUMO1 | - |  |
| troglitazone | TP53 | 29.8493 | Increased proliferation and decreased apoptosis of pulmonary artery smooth muscle cells are the main causes of hypoxic pulmonary hypertension. Troglitazone increases the apoptosis of the smooth muscle cells under hypoxic conditions by upregulating the PTEN expression through the PPARγ signaling pathway (Pi et al. 2013). |
| valproic acid | TP53 | 68.59 | Valproic acid was found to increase nitric oxide production by inhibiting the CDK5-Tyr(15)-eNOS-Ser(116) phosphorylation axis; thus it may be useful in the treatment of NO-related cerebro-cardiovascular diseases (Cho et al. 2014). |
|  | SUMO1 | - |  |

# References

Barnes GE, Bishop VS, and Kardon MB. 1972. Cardiac and peripheral vascular effects of pralidoxime chloride. *Eur J Pharmacol* 20:188-194.

Cho DH, Park JH, Joo Lee E, Jong Won K, Lee SH, Kim YH, Hwang S, Ja Kwon K, Young Shin C, Song KH, Jo I, and Han SH. 2014. Valproic acid increases NO production via the SH-PTP1-CDK5-eNOS-Ser(116) signaling cascade in endothelial cells and mice. *Free Radic Biol Med* 76:96-106. 10.1016/j.freeradbiomed.2014.07.043

Deng L, Hong T, Lin J, Ding S, Huang Z, Chen J, Jia J, Zou Y, Wang TC, Yang X, and Ge J. 2015. Histamine deficiency exacerbates myocardial injury in acute myocardial infarction through impaired macrophage infiltration and increased cardiomyocyte apoptosis. *Sci Rep* 5:13131. 10.1038/srep13131

Gasparotto J, Somensi N, Kunzler A, Girardi CS, de Bittencourt Pasquali MA, Ramos VM, Simoes-Pires A, Quintans-Junior LJ, Branco A, Moreira JC, and Gelain DP. 2014. Hecogenin acetate inhibits reactive oxygen species production and induces cell cycle arrest and senescence in the A549 human lung cancer cell line. *Anticancer Agents Med Chem* 14:1128-1135.

Jutkiewicz EM, Rice KC, Carroll FI, and Woods JH. 2013. Patterns of nicotinic receptor antagonism II: cardiovascular effects in rats. *Drug Alcohol Depend* 131:284-297. 10.1016/j.drugalcdep.2012.12.021

Marikovsky M, Nevo N, Vadai E, and Harris-Cerruti C. 2002. Cu/Zn superoxide dismutase plays a role in angiogenesis. *Int J Cancer* 97:34-41.

Mark-Kappeler CJ, Martin DS, and Eyster KM. 2011. Estrogens and selective estrogen receptor modulators regulate gene and protein expression in the mesenteric arteries. *Vascul Pharmacol* 55:42-49. 10.1016/j.vph.2011.05.002

Olejarz W, Bryk D, and Zapolska-Downar D. 2014. Mycophenolate mofetil--a new atheropreventive drug? *Acta Pol Pharm* 71:353-361.

Pi WF, Guo XJ, Su LP, and Xu WG. 2013. Troglitazone upregulates PTEN expression and induces the apoptosis of pulmonary artery smooth muscle cells under hypoxic conditions. *Int J Mol Med* 32:1101-1109. 10.3892/ijmm.2013.1487

Tan SM, Deliyanti D, Figgett WA, Talia DM, de Haan JB, and Wilkinson-Berka JL. 2015. Ebselen by modulating oxidative stress improves hypoxia-induced macroglial Muller cell and vascular injury in the retina. *Exp Eye Res* 136:1-8. 10.1016/j.exer.2015.04.015

Yin H, Gui Y, and Zheng XL. 2010. 2-methoxyestradiol inhibits atorvastatin-induced rounding of human vascular smooth muscle cells. *J Cell Physiol* 222:556-564. 10.1002/jcp.21970
