# Supplementary material for: Identify potential drugs for cardiovascular diseases caused by stress-induced genes in vascular smooth muscle cells
Source: PeerJ. 2016 Sep 28;4:e2478. doi: 10.7717/peerj.2478 (PMC5045879; doi:10.7717/peerj.2478)
Supplement: Supplemental Information 10 [file peerj-04-2478-s010.docx]

Table S1. A list of up-regulated DEGs.

| *ADGRL4* | *AHNAK* | *AKT3* | *AMD1* | *AOC4P* | *ARHGEF12* | *ARMCX2* |
| --- | --- | --- | --- | --- | --- | --- |
| *ARMCX6* | *ASAH1* | *ASPH* | *ATP6V0E1* | *AZIN1* | *BAG3* | *BIN3* |
| *BTG3* | *C7orf55-LUC7L2* | *CACNA1F* | *CCDC51* | *CCL2* | *CCL20* | *CD163* |
| *CD164* | *CDH15* | *CDH2* | *CDK19* | *CDKN2A* | *CEACAM1* | *CERS4* |
| *CHD3* | *CHODL* | *CKLF* | *CNTNAP2* | *CREBL2* | *CRY1* | *CTNND1* |
| *CXorf40B* | *CYB5R4* | *CYP51A1* | *DAB2* | *DICER1* | *DKC1* | *DNAJA1* |
| *DNMBP* | *DOHH* | *DPY19L4* | *DRAM1* | *DSCR4* | *DVL3* | *EHD1* |
| *EIF4A2* | *ELAVL3* | *ENTPD7* | *EPO* | *ESPL1* | *ETNK2* | *FABP2* |
| *FAM57A* | *FAS* | *FGF2* | *FNDC4* | *FOXA2* | *FST* | *GABRA4* |
| *GEMIN4* | *GM2A* | *GNL3* | *GNPAT* | *GOLPH3L* | *GPR161* | *GPRC5A* |
| *GRAP* | *GRPEL1* | *GRWD1* | *HADH* | *HAPLN2* | *HIST1H2BC* | *HIST1H2BE* |
| *HIST1H2BF* | *HIST1H2BG* | *HIST1H2BI* | *HMGCS1* | *HRH4* | *HS2ST1* | *HSPA1A* |
| *HSPA1B* | *HSPA1L* | *HSPB7* | *HSPH1* | *IDH3B* | *IGHG1* | *IL1B* |
| *IL7R* | *INO80B* | *INSR* | *KIAA0226L* | *KIAA0907* | *KIF15* | *KPNA2* |
| *LMAN2L* | *LMBRD1* | *LOC100129518* | *LOC100996724* | *LOC100996761* | *LOC100996792* | *LOC101928189* |
| *LPCAT4* | *LRRD1* | *LUC7L2* | *LUC7L3* | *LYPLA2* | *MAP2K3* | *MAP3K4* |
| *MAPRE2* | *MAT2A* | *MATN1* | *MCTP2* | *ME1* | *MEGF9* | *MIR1248* |
| *MIR1292* | *MIR4745* | *MIR664B* | *MLF2* | *MMD* | *MMP10* | *MRPL52* |
| *MTRR* | *MYL10* | *NCALD* | *NCAPH* | *NEFM* | *NEU1* | *NFATC2IP* |
| *NGF* | *NOLC1* | *NOP2* | *NOP56* | *NPC1* | *NPPC* | *NT5DC3* |
| *NUPL1* | *OAS2* | *OR10H3* | *PDE4DIP* | *PDGFB* | *PDLIM4* | *PDXK* |
| *PIGO* | *PKD2* | *PMCH* | *PNPLA2* | *PPAT* | *PPIF* | *PPP3CA* |
| *PRPF3* | *PRPF4* | *PSPC1* | *PTBP1* | *PTCRA* | *RBM25* | *RBPMS* |
| *RCBTB2* | *REG3A* | *RFC5* | *RNASEH1* | *RRS1* | *RSRP1* | *S100A5* |
| *SATB1* | *SDAD1* | *SDCBP* | *SGCE* | *SKP2* | *SLC22A7* | *SLC25A22* |
| *SLC39A8* | *SLC5A3* | *SMARCA5* | *SMOX* | *SMTN* | *SNORA4* | *SNORA56* |
| *SNORA63* | *SNORA81* | *SNORD110* | *SNORD19B* | *SNORD2* | *SNORD57* | *SNORD86* |
| *SOCS2* | *SOD2* | *SON* | *SQSTM1* | *SRR* | *SSX3* | *ST3GAL1* |
| *ST8SIA4* | *STAT1* | *STAT2* | *STATH* | *TBX5* | *TCL6* | *THOC1* |
| *TM4SF1* | *TMEM14A* | *TMX2-CTNND1* | *TNPO3* | *TPP1* | *TRIM27* | *TRIM31* |
| *TRIM32* | *TTC12* | *TUBGCP4* | *TULP2* | *TUSC3* | *WSB2* | *ZBTB11* |
| *ZDHHC18* | *ZNF189* | *ZNF318* | *ZNF467* | *ZSCAN32* | *ZYX* |  |
