# Supplementary material for: Identify potential drugs for cardiovascular diseases caused by stress-induced genes in vascular smooth muscle cells
Source: PeerJ. 2016 Sep 28;4:e2478. doi: 10.7717/peerj.2478 (PMC5045879; doi:10.7717/peerj.2478)
Supplement: Supplemental Information 11 [file peerj-04-2478-s011.docx]

Table S2. A list of down-regulated DEGs.

| *2-Mar* | *7-Mar* | *ABCA1* | *ACTL6A* | *ADGRG1* | *ADRB2* | *AGL* |
| --- | --- | --- | --- | --- | --- | --- |
| *AHNAK2* | *AKAP13* | *AKAP9* | *AMIGO2* | *AMOTL2* | *ANKRD11* | *ARHGAP35* |
| *ARHGDIA* | *ARL4A* | *ARMC9* | *ASF1A* | *ASS1* | *ATAD2* | *ATF5* |
| *ATP5D* | *B2M* | *BAX* | *BAZ2B* | *BBX* | *BCL2L1* | *BCL6* |
| *BHLHE40* | *BLVRA* | *BMPR1A* | *C10orf76* | *C14orf1* | *C20orf27* | *CALHM2* |
| *CALM1* | *CALM2* | *CALM3* | *CAMSAP2* | *CAV2* | *CCDC91* | *CCNG2* |
| *CCNL1* | *CCP110* | *CCPG1* | *CDC42BPA* | *CDC42EP3* | *CDCP1* | *CDH13* |
| *CDK2* | *CDK5RAP3* | *CDS2* | *CEP170* | *CEP170P1* | *CEP350* | *CEP55* |
| *CEP57* | *CEP76* | *CFLAR* | *CHAF1B* | *CITED2* | *CLASP1* | *CLCC1* |
| *CLIC4* | *CLN5* | *CNOT2* | *CPQ* | *CRBN* | *CREBL2* | *CRTC3* |
| *CRYZ* | *CTGF* | *CUL5* | *CXCL2* | *CYB5B* | *CYB5R1* | *CYR61* |
| *DAAM1* | *DEK* | *DHX40* | *DIAPH2* | *DKK2* | *DLC1* | *DNASE2* |
| *DPYD* | *DUSP1* | *DYX1C1-CCPG1* | *ECSIT* | *ECT2* | *EGFR* | *EGR2* |
| *EGR3* | *ELP4* | *ENC1* | *ENOSF1* | *EPRS* | *ERMAP* | *ETV5* |
| *EXOC2* | *EXOSC8* | *F2R* | *FAM149B1* | *FAM172A* | *FAM20B* | *FAM21C* |
| *FAM69A* | *FANCG* | *FBXO16* | *FBXW11* | *FCHSD2* | *FEM1B* | *FKBP1B* |
| *FMR1* | *FOSB* | *FPGT* | *FRYL* | *FSCN1* | *GALNT10* | *GANAB* |
| *GCOM1* | *GLTSCR1L* | *GNS* | *GPBP1L1* | *GPD1L* | *GTPBP2* | *GULP1* |
| *H2AFY* | *HBS1L* | *HDGFRP3* | *HECA* | *HECTD4* | *HHEX* | *HHLA3* |
| *HIBCH* | *HIST1H1C* | *HMGB1* | *HMGB3* | *HMGXB4* | *HNRNPD* | *HSPG2* |
| *ID4* | *IFI16* | *IFNGR1* | *IFT74* | *IL1A* | *IL23A* | *IRF9* |
| *JUNB* | *JUP* | *KAT5* | *KAT6B* | *KCNJ2* | *KDR* | *KIAA0355* |
| *KIAA1109* | *KLF4* | *KLF9* | *KLHL22* | *KLHL24* | *KLHL7* | *LARP6* |
| *LGR5* | *LINC00623* | *LINC00869* | *LMO4* | *LRRC1* | *LRRC49* | *LYRM1* |
| *MACF1* | *MAP3K8* | *MARCKS* | *MBNL2* | *MDC1* | *MED14* | *MFAP4* |
| *MGEA5* | *MIR4680* | *MIR6787* | *MKL2* | *MKNK2* | *MKRN1* | *MMP14* |
| *MOCOS* | *MRE11A* | *MSC* | *MTHFD1* | *MTMR3* | *MUM1* | *MYRIP* |
| *NBN* | *NCAPG2* | *NCK2* | *NCOR1* | *NDUFB7* | *NET1* | *NLRP1* |
| *NMI* | *NMRK1* | *NPAS2* | *NPEPPS* | *NR2F1* | *NR2F2* | *NR4A1* |
| *NR4A2* | *NRG1* | *NSL1* | *NT5C* | *NTAN1* | *NTM* | *NUDT9* |
| *NUMA1* | *NUP133* | *NUP155* | *NUPR1* | *OBFC1* | *OIP5* | *PALMD* |
| *PANK4* | *PAPOLA* | *PARP8* | *PAWR* | *PCBP4* | *PCIF1* | *PCMTD2* |
| *PCYOX1* | *PDCD4* | *PDLIM7* | *PDP1* | *PDS5A* | *PFDN4* | *PHACTR4* |
| *PHB* | *PHF21A* | *PHF3* | *PHKB* | *PICALM* | *PLAU* | *PLCL1* |
| *PLEC* | *PLIN2* | *PLSCR1* | *PMAIP1* | *PMS1* | *POLI* | *POLR2M* |
| *POMGNT1* | *PORCN* | *PPAP2B* | *PPM1A* | *PPM1D* | *PPP1CB* | *PPP2R3C* |
| *PPWD1* | *PRKD3* | *PRPSAP2* | *PSD3* | *PSPH* | *PSPHP1* | *PTEN* |
| *PTGER4* | *PTPRF* | *PUM2* | *RAB3GAP1* | *RAB4A* | *RAD21* | *RAD50* |
| *RALBP1* | *RARA* | *RB1CC1* | *RBAK* | *RBFOX2* | *RBPMS* | *RCAN1* |
| *RCBTB1* | *RCC1* | *REV3L* | *RFC1* | *RFC4* | *RFXANK* | *RGL1* |
| *RGS4* | *RHOB* | *RHOBTB3* | *RHOT1* | *RNF126* | *RNF41* | *ROBO1* |
| *RSF1* | *RXRA* | *SARDH* | *SAV1* | *SCAF11* | *SDC2* | *SGCB* |
| *SGK1* | *SHB* | *SIK1* | *SLC16A3* | *SLC17A9* | *SLC1A4* | *SLC25A12* |
| *SLC26A2* | *SLC35A5* | *SMARCE1* | *SMPD1* | *SNAP23* | *SNX13* | *SNX2* |
| *SOCS5* | *SOX4* | *SP100* | *SP110* | *SPHAR* | *SPTBN1* | *SRPK1* |
| *SRSF5* | *STAG2* | *STAT1* | *STAT6* | *STK3* | *STX16* | *SULF1* |
| *SUMO1* | *SUPT7L* | *SUZ12* | *SUZ12P1* | *SYT11* | *TBC1D22A* | *TBC1D2B* |
| *TBX2* | *TCAF1* | *TCF4* | *TCF7L2* | *TES* | *TIA1* | *TMEM123* |
| *TMEM242* | *TMEM35* | *TMEM38B* | *TNFAIP6* | *TP53* | *TP53I11* | *TRAK1* |
| *TRBV19* | *TRIB2* | *TRIB3* | *TRIM2* | *TSPAN13* | *TTLL7* | *UBE2W* |
| *UBR5* | *UGCG* | *USP46* | *UVRAG* | *VCAN* | *VGLL4* | *VIPR1* |
| *VRK1* | *WBP1L* | *WDR11* | *WEE1* | *WIPF1* | *WSB1* | *WWTR1* |
| *XPO1* | *YKT6* | *YWHAE* | *ZBTB20* | *ZEB1* | *ZFAND5* | *ZFP36L1* |
| *ZMYM4* | *ZNF292* | *ZNF32* | *ZNF331* | *ZNF395* | *ZNF668* |  |
