# Supplementary material for: Identify potential drugs for cardiovascular diseases caused by stress-induced genes in vascular smooth muscle cells
Source: PeerJ. 2016 Sep 28;4:e2478. doi: 10.7717/peerj.2478 (PMC5045879; doi:10.7717/peerj.2478)
Supplement: Supplemental Information 12 [file peerj-04-2478-s012.docx]

Table S3. The top two largest up- (down-) regulated clusters with sizes 7 and 6 (22 and 8) after ClusterONE analysis.

| Up-regulated cluster (7 DEGS) | | | | | | | |
| --- | --- | --- | --- | --- | --- | --- | --- |
| *CDKN2A* | *GNL3* | *GPRC5A* | *NOLC1* | *NOP2* | *NOP56* | *RRS1* |  |
| Up-regulated cluster (6 DEGS) | | | | | | | |
| *BAG3* | *DOHH* | *EIF4A2* | *HSPA1A* | *HSPA1L* | *MLF2* |  |  |
| Down-regulated cluster (22 DEGS) | | | | | | | |
| *ACTL6A* | *C20orf27* | *CFLAR* | *HMGB1* | *KAT5* | *KLF4* | *NR4A1* | *PDLIM7* |
| *PTEN* | *RCC1* | *RSF1* | *SMARCE1* | *SOX4* | *SP100* | *SUMO1* | *SUPT7L* |
| *TCF4* | *TP53* | *UBR5* | *VRK1* | *ZEB1* | *ZNF668* |  |  |
| Down-regulated cluster (8 DEGS) | | | | | | | |
| *FBXW11* | *KDR* | *PDCD4* | *PFDN4* | *RCAN1* | *SHB* | *WEE1* | *ZNF395* |
