# Supplementary material for: Identify potential drugs for cardiovascular diseases caused by stress-induced genes in vascular smooth muscle cells
Source: PeerJ. 2016 Sep 28;4:e2478. doi: 10.7717/peerj.2478 (PMC5045879; doi:10.7717/peerj.2478)
Supplement: Supplemental Information 13 [file peerj-04-2478-s013.docx]

Table S4. The results of IC_liver_50 values for three liver cancer cell lines, including Mahlavu, Huh7, and PLC5 cells. The table summarized the IC_liver_50 values after the treatment of 72 hours.

| Drug | Annotation | IC_liver_50 values of 72h (μM) | | |
| --- | --- | --- | --- | --- |
|  |  | Mahlavu | HuH7 | PLC5 |
| lomustine | Chemotherapy for brain tumor | - | - | - |
| parthenolide | Potential proteasome inhibitor/ NF-kB/ HSP inhibitor | 1~3.3 | > 10 | - |
| phenoxybenzamine | Alpha-receptor antagonist for hypertension | > 10 | >10 | >10 |
| piperlongumine | Modulator of ROS levels; Potential anti-neoplastics | 0.33~1 | 3.3~10 | 3.3~10 |
| securinine | Selective GABA receptor antagonist | ~10 | >10 | >10 |
| sulconazole | Anti-fungal agent | > 10 | >10 | >10 |
| tanespimycin | HSP90 inhibitor | - | - | - |
| thiostrepton | FOXM1 inhibitor/ Cancer stem cell inhibitor/ Potent HSP inhibitor | 1~3.3 | 3.3~10 | >10 |
| trifluoperazine | Anti-psychotic agent/ Cancer stem cell inhibitor/ Autophagy | >10 | >10 | >10 |
| vorinostat | HDAC inhibitor | 3.3~10 | 1~3.3 | >10 |

‘-‘ denotes not determined
