# Supplementary material for: Identify potential drugs for cardiovascular diseases caused by stress-induced genes in vascular smooth muscle cells
Source: PeerJ. 2016 Sep 28;4:e2478. doi: 10.7717/peerj.2478 (PMC5045879; doi:10.7717/peerj.2478)
Supplement: Supplemental Information 14 [file peerj-04-2478-s014.docx]

Table S5. The results of cMAP drugs, their target genes (DEG), the IC_db_50 values, and potential (cardio)vascular-acting mechanisms on CVDs information.

| **Drug name** | **Target gene (DEG)** | **IC_db_50 (μM)** | **Potential (cardio)vascular-acting mechanisms on CVD** |
| --- | --- | --- | --- |
| ***No study found yet on CVD/VSMC acting mechanisms.*** | | | |
| alpha-ergocryptine | TP53 | - | No study found on CVD/VSMC. |
|  | SUMO1 | - |  |
| carmustine | TP53 | 1.188 | No study found on CVD/VSMC. |
|  | SUMO1 | - |  |
| chlorambucil | HSPA1A | - | No study found on CVD/VSMC. |
|  | TP53 | 22.39 |  |
|  | SUMO1 | - |  |
| cyclacillin | TP53 | - | No study found on CVD/VSMC. |
| dienestrol | HSPA1A |  | No study found on CVD/VSMC. |
|  | TP53 | 3.916 |  |
|  | SUMO1 | - |  |
| flutamide | HSPA1A | - | No study found on CVD/VSMC. |
|  | TP53 | 73.31 |  |
|  | SUMO1 | - |  |
| hexestrol | TP53 | - | No study found on CVD/VSMC. |
|  | SUMO1 | - |  |
| isoxicam | HSPA1A | - | No study found on CVD/VSMC. |
|  | TP53 | - |  |
|  | SUMO1 | - |  |
| rifabutin | TP53 | 59.77 | No study found on CVD/VSMC. |
|  | SUMO1 | - |  |
| semustine | TP53 | - | No study found on CVD/VSMC. |
|  | SUMO1 | - |  |
| ***Potential effects acting on CVD/VSMC with demonstrated mechanisms.*** | | | |
| cimetidine | HSPA1A | - | Results of in vivo and in vitro assays showed that histamine deficiency promotes cardiomyocytes apoptosis. Administering cimetidine demonstrated a protective effect of histamine against myocardial injury (Deng et al. 2015). |
|  | TP53 | 60.57 |  |
|  | SUMO1 | - |  |
| cytisine | TP53 | - | A nicotinic receptor agonist. The cardiovascular effects are due to activation at autonomic ganglia involving nicotinic receptor subtypes other than α4, α7, or β2 (Jutkiewicz et al. 2013). |
|  | SUMO1 | - |  |
| disulfiram | HSPA1A | - | Oral administration of disulfiram, an inhibitor of Cu/Zn superoxide dismutase (SOD), inhibited angiogenesis in Tg-SOD mice as well as in CD1 nude mice (Marikovsky et al. 2002). |
|  | TP53 | 25.12 |  |
|  | SUMO1 | - |  |
| ebselen | HSPA1A | - | Ebselen may be potential treatments for retinopathies that feature oxidative stress-mediated damage to glia and the microvasculature (Tan et al. 2015). |
|  | TP53 | 7.943 |  |
|  | SUMO1 | - |  |
| equilin | HSPA1A | - | Upregulation of SCD gene expression  with equilin treatment may contribute to a possible protective role of this estrogen in  the mesenteric arteries (Mark-Kappeler et al. 2011). |
|  | TP53 | 76.96 |  |
|  | SUMO1 | - |  |
| hecogenin | TP53 | - | Hecogenin acetate is able to modulate reactive species production, inducing cell cycle arrest and senescence and also modulating ERK1/2 phosphorylation and MMP-2 production (Gasparotto et al. 2014). |
| monastrol | TP53 | - | A kinesin Eg5 inhibitor. Kinesins are known to perform mechanical work to power a variety of cellular functions, from mitosis to organelle transport (Yin et al. 2010). |
| mycophenolic acid | TP53 | 0.06766 | Mycophenolate mofetil has anti-atherogenic effects at the level of endothelial cells, monocytes/ macrophages, smooth muscle cells and dendritic cells. It also exhibits anti-oxidative properties (Olejarz et al. 2014). |
|  | SUMO1 | - |  |
| pralidoxime | HSPA1A | - | Cardiac and peripheral vascular effects of pralidoxime chloride (Barnes et al. 1972). |
|  | TP53 | - |  |
|  | SUMO1 | - |  |
| troglitazone | TP53 | 29.8493 | Increased proliferation and decreased apoptosis of pulmonary artery smooth muscle cells are the main causes of hypoxic pulmonary hypertension. Troglitazone increases the apoptosis of the smooth muscle cells under hypoxic conditions by upregulating the PTEN expression through the PPARγ signaling pathway (Pi et al. 2013). |
| valproic acid | TP53 | 68.59 | Valproic acid was found to increase nitric oxide production by inhibiting the CDK5-Tyr(15)-eNOS-Ser(116) phosphorylation axis; thus it may be useful in the treatment of NO-related cerebro-cardiovascular diseases (Cho et al. 2014). |
|  | SUMO1 | - |  |
